# Methanol‐Ethanol Discrimination and Selective Sensing Enabled by Molecular Sieving in Conductive MOFs

**DOI:** 10.1002/adma.73406

**Published:** 2026-05-19

**Authors:** Young‐Moo Jo, Mingyu Jeon, Dong‐Ha Kim, Jihan Kim, Mircea Dincă

**Affiliations:** ^1^ Department of Chemistry Massachusetts Institute of Technology Cambridge Massachusetts USA; ^2^ School of Materials Science and Engineering Kyungpook National University Daegu Republic of Korea; ^3^ Department of Chemical and Biomolecular Engineering Korea Advanced Institute of Science and Technology Daejeon Republic of Korea; ^4^ Department of Materials Science and Chemical Engineering Hanyang University Ansan Republic of Korea; ^5^ Department of Chemistry Princeton University Princeton New Jersey USA

**Keywords:** carbon nanotube, chemiresistor, conductive metal‐organic frameworks, methanol, molecular sieving

## Abstract

Methanol presents a significant health risk because of its volatility and toxicity. Its close chemical similarity to ethanol increases the likelihood of accidental ingestion through contaminated beverages. Here, we report chemiresistive sensors that selectively distinguish methanol from ethanol under ambient conditions. The sensors consist of single‐walled carbon nanotubes (CNTs) functionalized with conductive metal–organic frameworks (cMOFs) constructed from 2,3,7,8,12,13‐hexahydroxytetraazanaphthotetraphene (HHTT). Trinuclear intra‐pore clusters (IPCs) located within the honeycomb channels of HHTT‐based cMOFs govern methanol sensing by increasing the density of adsorption sites and constraining molecular diffusion through the pores. Mg‐HHTT, in which the pores are largely occupied by IPCs, enhances the methanol response of CNT@cMOF composites while suppressing transport of ethanol and larger alcohols. In contrast, isostructural Ni‐HHTT and Cu‐HHTT analogues, which lack a high density of IPCs, exhibit substantially lower sensitivity and selectivity. Density functional theory and molecular dynamics simulations support a sensing mechanism based on IPC‐mediated molecular sieving. Sensor tests using methanol‐spiked liquors demonstrate selective methanol detection in complex beverage matrices under practical ambient conditions.

## Graphical abstract

Selective methanol sensors are achieved by coating carbon nanotube chemiresistors with two dimensional conductive metal–organic frameworks that incorporate intra‐pore clusters. These confined clusters increase the density of adsorption sites while restricting the transport of larger interfering molecules, particularly ethanol. This sensing architecture enables sensitive and selective detection of methanol in alcoholic beverages under realistic conditions.

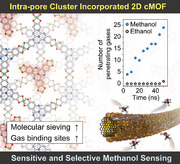

## 1 Introduction

Methanol is widely used as an industrial solvent in applications ranging from fuel production to the manufacture of plastics and synthetic fibers, and is also present in consumer products such as antifreeze [1]. Despite its widespread use, methanol is also a potent poison. Upon ingestion, hepatic metabolism converts methanol into formaldehyde and formic acid, leading to severe damage to the nervous, vision, and digestive systems and, in some cases, fatal outcomes [2, 3]. Methanol's close chemical similarity to ethanol increases the risk of accidental ingestion through contaminated alcoholic beverages, and poisoning incidents, including fatalities, continue to be reported worldwide [4, 5]. Although gas chromatography–mass spectrometry can reliably distinguish methanol from ethanol [6], the method‘s cost, size, operational complexity, and analysis time limit its suitability for routine or consumer‐level screening.

Chemiresistive gas sensors provide a low‐cost alternative to spectroscopic techniques because of their simple operating principles [7]. Recent advances in carbon nanotube (CNT)‐based sensors have enabled room‐temperature operation without external heating, thereby reducing energy consumption and supporting integration into portable devices [8]. Despite these advances, CNT‐based sensors typically exhibit limited sensitivity and selectivity, which arises from the low density of chemically active surface sites available for gas adsorption. Consequently, surface functionalization has been widely explored to increase analyte–surface interactions, with demonstrated improvements for gases such as methane and hydrogen sulfide [9, 10]. In contrast, sensors capable of efficiently and selectively discriminating between methanol and ethanol remain limited. Here, we report a chemiresistive sensor that distinguishes methanol from ethanol at room temperature.

Selective gas sensing can be achieved through the use of filtering layers that separate analytes based on diffusivity and kinetic diameter rather than chemical reactivity [11, 12]. Effective filtration requires materials with narrowly distributed pore sizes spanning the angstrom‐to‐nanometer regime. Metal–organic frameworks (MOFs) are well suited for this role because their crystalline order and modular structures enable precise control over pore dimensions [13, 14, 15, 16]. A central limitation of filtration‐based selectivity is that diffusion barriers can reduce the flux of the target analyte to the active sensing surface, thereby diminishing sensitivity. To address this constraint, we sought to design a filtering layer for CNT‐based sensors that discriminates against interferents on the basis of molecular size while preserving analyte access and increasing the density of reactive sites available for interaction with the target molecule.

On this basis, we developed a methanol sensor with enhanced sensitivity and selectivity by coating CNT chemiresistors with two dimensional conductive metal–organic frameworks (cMOFs) based on Mg‐HHTT (HHTT = 2,3,7,8,12,13‐hexahydroxytetraazanaphthotetraphene). Mg‐HHTT incorporates several structural features relevant to methanol sensing, including heterocyclic nitrogen atoms that resemble active sites in nitrogen‐doped carbon materials [17, 18], Mg^2+^ nodes bearing exchangeable solvent ligands that can function as inner‐sphere binding sites for alcohols [19], and discrete Mg_3_HHTT IPCs in which each Mg^2+^ center coordinates multiple solvent molecules. These clusters occupy a large fraction of the pore volume, thereby constraining diffusion of larger molecules such as ethanol. In addition, the intrinsic electrical conductivity of Mg‐HHTT enables direct transduction of adsorption‐induced charge perturbations to the underlying CNT network, in contrast to insulating MOF coatings that primarily act as passive analyte concentrators [20, 21]. Comparison with other HHTT‐based cMOFs, including Ni‐HHTT with dispersed IPCs and Cu‐HHTT lacking such clusters, highlights the role of intra‐pore structure in governing sensitivity and selectivity. Density functional theory (DFT) calculations show that methanol binds more strongly to Mg‐HHTT than to the corresponding Ni‐HHTT or Cu‐HHTT frameworks. Molecular dynamics (MD) simulations further indicate that IPCs in Mg‐HHTT preferentially permit methanol transport while restricting ethanol diffusion. Consistent with these predictions, sensor measurements using methanol‐spiked alcoholic beverages demonstrate the functional advantages of cMOF‐coated CNT chemiresistors for selective methanol detection.

## 2 Result and Discussion

### 2.1 Preparation of Carbon Nanotube@Conductive Metal‐Organic Frameworks Composites

**FIGURE 1:**
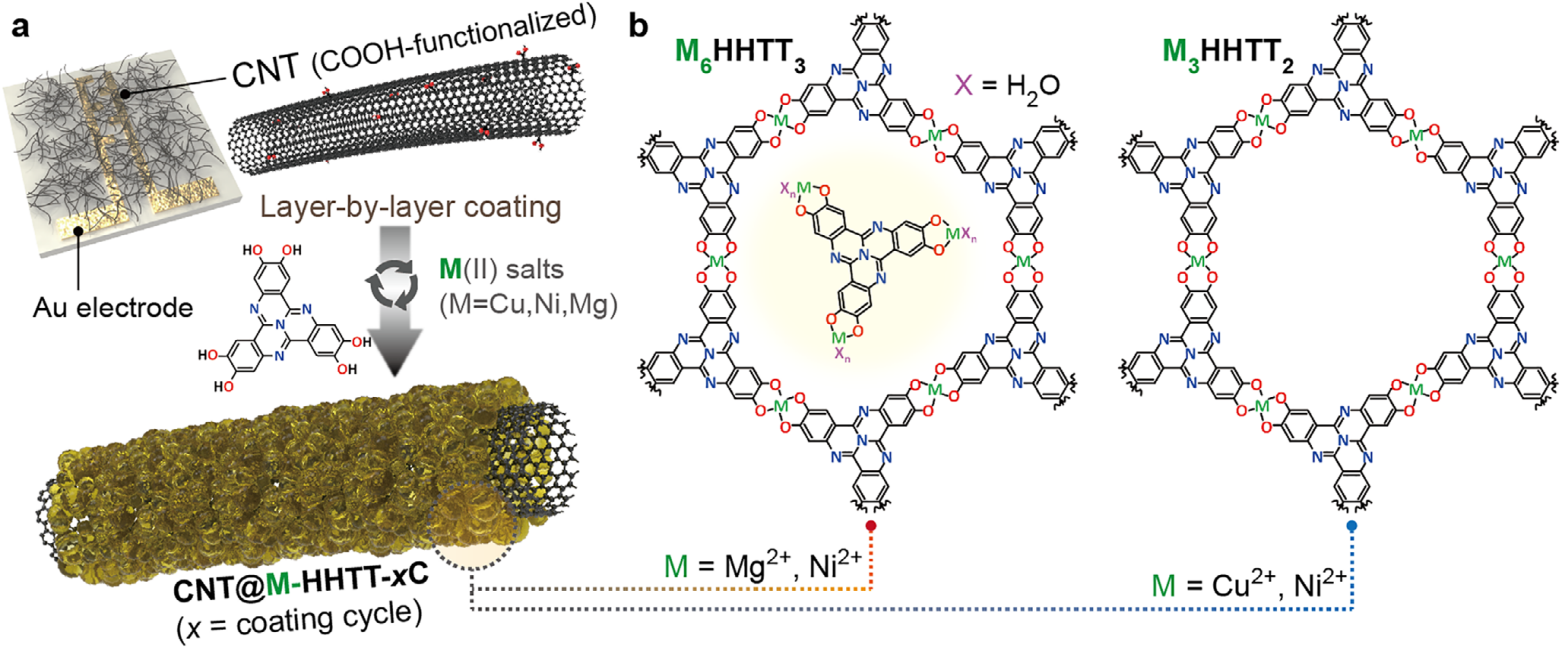
(a) Schematic representation of the fabrication procedure for M‐HHTT (M = Cu, Ni, Mg)–coated CNT sensors. (b) Partial structural representations of M_6_HHTT_3_ (M = Mg, Ni; left) and M_3_HHTT_2_ (M = Cu, Ni; right).

Figure 1a outlines the fabrication of CNT chemiresistors coated with M‐HHTT (M = Cu, Ni, Mg). Carboxylic acid (COOH^−^)–functionalized single‐walled CNTs were deposited onto Au interdigitated electrode/alumina substrates by drop‐casting. Deposition from a dilute CNT slurry (0.05 mg/mL) yields a low‐density network that facilitates gas diffusion throughout the sensing layer, as confirmed by scanning electron microscope (SEM) (Figure S1). The CNTs form a well‐percolated network that serves as the primary conductive pathway. Surface carboxylate groups promote the adsorption of metal ions and act as nucleation sites for the subsequent growth of conductive MOF layers. M‐HHTT coatings were formed by layer‐by‐layer (LBL) deposition, in which CNT substrates were alternately immersed in ethanol solutions containing M(II) salts (1 mm; M = Cu, Ni, Mg) and H_6_HHTT (0.1 mm) for 2 and 4 min, respectively. After each immersion, the substrates were rinsed with ethanol to remove residual salts and suppress inhomogeneous or excessive cMOF growth. The resulting devices are denoted CNT@M‐HHTT‐*x*C, where M indicates the metal center (M = Cu, Ni, Mg) and *x* denotes the number of growth cycles (*x* = 1–15). For comparison, CNT@Cu‐HHTP‐*x*C sensors (HHTP = 2,3,6,7,10,11‐hexahydroxytriphenylene; *x* = 1–15) were prepared by substituting HHTT with HHTP to assess ligand‐dependent sensing behavior. SEM analysis shows a monotonic increase in coating thickness with increasing cycle number for Mg‐HHTT, Ni‐HHTT, and Cu‐HHTT layers (Figures S2–S4). TEM images further confirm complete encapsulation of CNTs (∼4 nm thickness) by Mg‐HHTT‐15C coatings (∼30 nm thickness) (Figure S5). As reported previously [22], HHTT‐based cMOFs adopt metal‐dependent phases (Figure 1b): Cu^2+^ forms Cu_3_HHTT_2_ structures composed exclusively of extended sheets, Mg^2+^ forms Mg_6_HHTT_3_ phases consisting of Mg_3_HHTT_2_ sheets and Mg_3_HHTT IPCs, and Ni^2+^ can generate both Ni_3_HHTT_2_ and Ni_6_HHTT_3_ phases. These structural differences are aligned with the coordination preferences of the metal nodes: whereas Cu^2+^ typically adopts a square‐planar geometry, Mg^2+^ prefers an octahedral coordination environment, and Ni^2+^ can accommodate both geometries.

**FIGURE 2:**
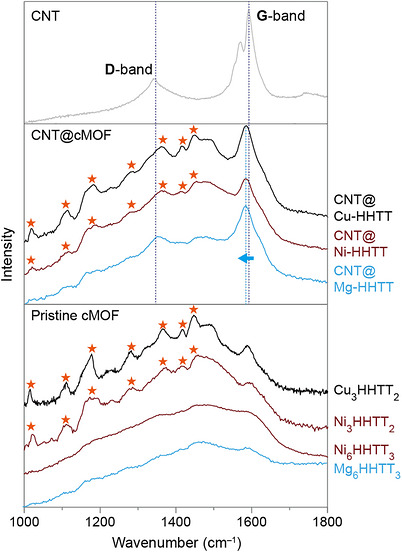
Raman spectra of pristine CNT, CNT@M‐HHTT‐15C, M_3_HHTT_2_ (M = Cu, Ni), and M_6_HHTT_3_ (M = Ni, Mg) powders.

The phases of CNT@M‐HHTT‐15C (M = Cu, Ni, Mg) were examined by Raman spectroscopy through comparison with reference spectra of pristine CNTs and phase‐pure M_3_HHTT_2_ (M = Cu, Ni) and M_6_HHTT_3_ (M = Mg, Ni) powders (Figure 2). Pristine CNTs exhibit characteristic D‐ and G‐band peaks at 1340 and 1593 cm^−1^, corresponding to defect‐induced and graphitic carbon vibrations, respectively [23]. Upon cMOF functionalization, the CNT G‐band shifts by 8 cm^−1^ to lower wavenumber, indicating electronic interaction between CNTs and the cMOFs, which act as n‐type dopants [8].

As reference materials, the corresponding cMOF powders were synthesized, and their powder X‐ray diffraction (PXRD) patterns are shown in Figures S6–S9. In the powder system, the presence or absence of IPCs in Ni‐HHTT can be selectively controlled by systematically tuning the synthesis conditions [22]. The Raman spectra of the cMOF reference powders show two distinct behaviors. Cu_3_HHTT_2_ and Ni_3_HHTT_2_, which lack IPCs, display multiple well‐defined peaks in the 1000–1600 cm^−1^ region, whereas Ni_6_HHTT_3_ and Mg_6_HHTT_3_, which incorporate IPCs, exhibit broader and less resolved features consistent with overlapping vibrational modes. The CNT@Cu‐HHTT‐15C spectrum closely matches that of Cu_3_HHTT_2_, confirming successful functionalization of extended‐sheet Cu_3_HHTT_2_ on CNTs. In contrast, CNT@Ni‐HHTT‐15C shows broadened and attenuated features relative to CNT@Cu‐HHTT‐15C, consistent with the coexistence of Ni_3_HHTT_2_ and Ni_6_HHTT_3_ phases. The CNT@Mg‐HHTT‐15C spectrum lacks discrete peaks and is dominated by broad features characteristic of the Mg_6_HHTT_3_ phase, indicating predominant formation of IPC‐containing Mg‐HHTT.

In addition, X‐ray photoelectron spectroscopy () analysis was conducted to confirm the composition of the cMOF layers. Clear peaks corresponding to Cu, Ni, and Mg were observed in CNT@Cu‐HHTT‐15C, CNT@Ni‐HHTT‐15C, and CNT@Mg‐HHTT‐15C samples, respectively, together with the N and O atoms of the HHTT ligand (Figures S10–S12).

### 2.2 Gas Sensing Characteristics of CNT@cMOF Composites

The gas sensing behavior of CNT@M‐HHTT‐*x*C sensors (M = Cu, Ni, Mg) toward methanol and representative interfering volatile organic compounds (VOCs), including ethanol, propanol, butanol, acetone, toluene, benzene, phenylethanol, and isoamyl alcohol, was evaluated at room temperature. Analyte concentrations were controlled using a VOC generator operated at defined temperatures and delivered in synthetic dry air (Figure S13). For each condition, at least three sensors were tested to ensure reproducibility. The resistance of pristine CNT and CNT@M‐HHTT‐xC sensors in air remained similar (1–10 kΩ), indicating that the primary electrical conduction pathway is maintained through the CNT network even after cMOF coating (Figure S14). Gas sensing measurements were conducted by exposing the sensors to analyte vapors for 10 min, followed by recovery in fresh air (Figures S15–S17). Sensor responses were quantified as *S*  =  (R_g_ − R_a_)/R_a_  ×  100%, where R_g_ and R_a_ denote the sensor resistance in the presence of analyte gas and in air, respectively (Figures S18–S20).

**FIGURE 3:**
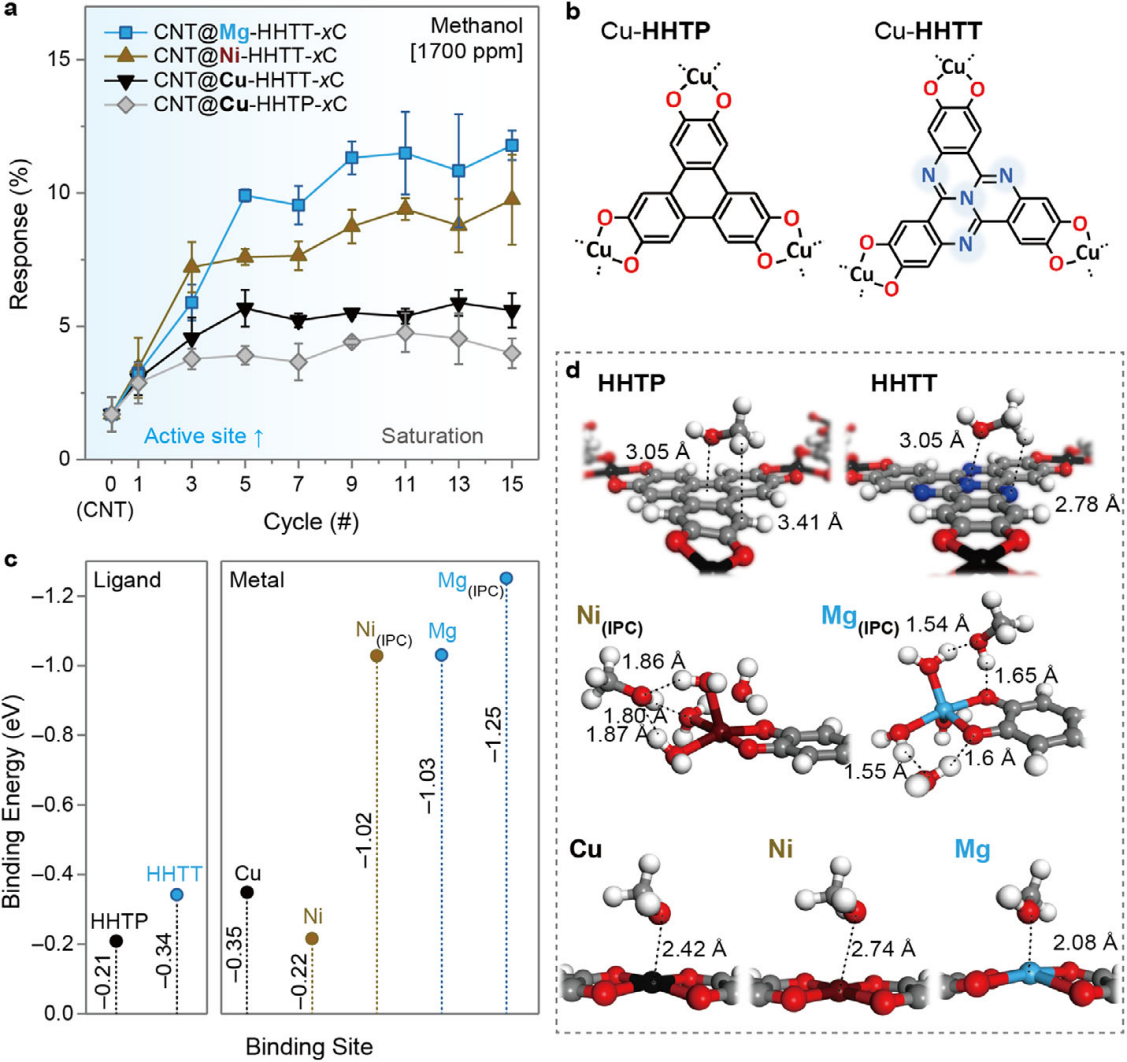
(a) Gas responses of CNT@M‐HHTT‐*x*C (M = Cu, Ni, Mg) and CNT@Cu‐HHTP‐*x*C sensors as a function of coating cycle number (*x* = 1–15). Error bars represent the standard deviation calculated from measurements of three independent sensors. (b) Chemical structures of Cu‐HHTP and Cu‐HHTT. (c) Density functional theory calculated binding energies and (d) corresponding binding configurations of methanol at representative binding sites in cMOFs (ligands: HHTP and HHTT; metal sites: Ni_(IPC)_, Mg_(IPC)_, Cu, Ni, and Mg).

Methanol responses for all sensors are summarized in Figure 3a to evaluate the effect of cMOF overlayers. Pristine CNT sensors exhibit a negligible methanol response (*S* = 1.69%), consistent with the absence of specific adsorption sites. Upon cMOF functionalization, the methanol response increases with coating thickness and reaches a maximum at approximately five deposition cycles. This enhancement reflects the introduction of additional analyte binding sites provided by the cMOF layers. At this thickness, CNT@Cu‐HHTP‐5C and CNT@Cu‐HHTT‐5C sensors display 2.3‐ and 3.6‐fold increases in methanol response, respectively, relative to pristine CNTs. Although Cu_3_HHTP_2_ and Cu_3_HHTT_2_ share analogous hexagonal frameworks with open pores, the higher response of CNT@Cu‐HHTT‐5C is attributed to the presence of pyridinic and graphitic nitrogen atoms in the HHTT linker (Figure 3b). These nitrogen sites act as additional adsorption centers in conjunction with CuO_4_ clusters, providing stronger analyte interactions than carbon‐only frameworks [24, 25]. DFT calculations support this interpretation, showing weaker methanol binding to the carbon framework of HHTP (−0.21 eV) compared to binding at nitrogen sites in HHTT (−0.34 eV) (Figure 3c; Ligand). The most stable configuration corresponds to methanol interacting with the nitrogen‐containing region of the linker, where the oxygen atom of methanol interacts with the graphitic nitrogen site at a distance of 3.05 Å, and the hydrogen atoms of the CH_3_ group interact with the pyridinic nitrogen site at a distance of 2.78 Å. The presence of nitrogen atoms in the HHTT framework introduces localized regions of high electron density, allowing the lone pair electrons to interact more strongly with methanol. Accordingly, the incorporation of nitrogen atoms in Cu‐HHTT increases the density and strength of methanol adsorption sites, resulting in enhanced sensing performance.

The influence of IPCs varies strongly with the identity of the metal center and is evident in the methanol sensing response. CNT@Cu‐HHTT‐5C, which lacks IPCs, and CNT@Ni‐HHTT‐5C, which contains dispersed IPCs, exhibit 3.6‐ and 4.5‐fold enhancements in methanol response, respectively, both lower than the 5.9‐fold enhancement observed for CNT@Mg‐HHTT‐5C, which contains saturated IPCs (Figure 3a). This trend indicates that IPCs increase methanol response by densifying reactive adsorption sites within the framework. In the M_6_HHTT_3_ phase, IPCs (molecular M_3_HHTT units) incorporate three metal centers and one ligand, doubling the number of metal sites and increasing the number of ligand nitrogen sites by 50% per unit cell relative to the IPC‐free M_3_HHTT_2_ structure.

To evaluate both site density and adsorption strength, methanol binding energies at distinct metal environments were calculated using DFT (Figure 3c,d). Methanol adsorption at extended‐layer MO_4_ clusters in Cu‐HHTT and Ni‐HHTT yields binding energies of −0.35 and −0.22 eV, respectively. In contrast, methanol binding at IPC metal sites in Ni‐HHTT exhibits a substantially stronger interaction of −1.02 eV. Although CuO_4_ clusters bind methanol more strongly than NiO_4_ clusters, the lower density of active sites in CNT@Cu‐HHTT‐*x*C limits its sensing response relative to CNT@Ni‐HHTT‐*x*C. These results indicate that IPCs enhance sensing performance by simultaneously increasing the number of adsorption sites and facilitating charge transfer to the conductive CNT network.

Mg‐HHTT exhibits particularly strong methanol binding, both at IPC sites (−1.25 eV) and at MgO_4_ clusters within extended layers (−1.03 eV). Combined with its high IPC density, this dual contribution provides both strong analyte interaction and a high concentration of active sites. Accordingly, DFT identifies Mg‐HHTT as the most effective coating among the HHTT‐based cMOFs for methanol sensing.

**FIGURE 4:**
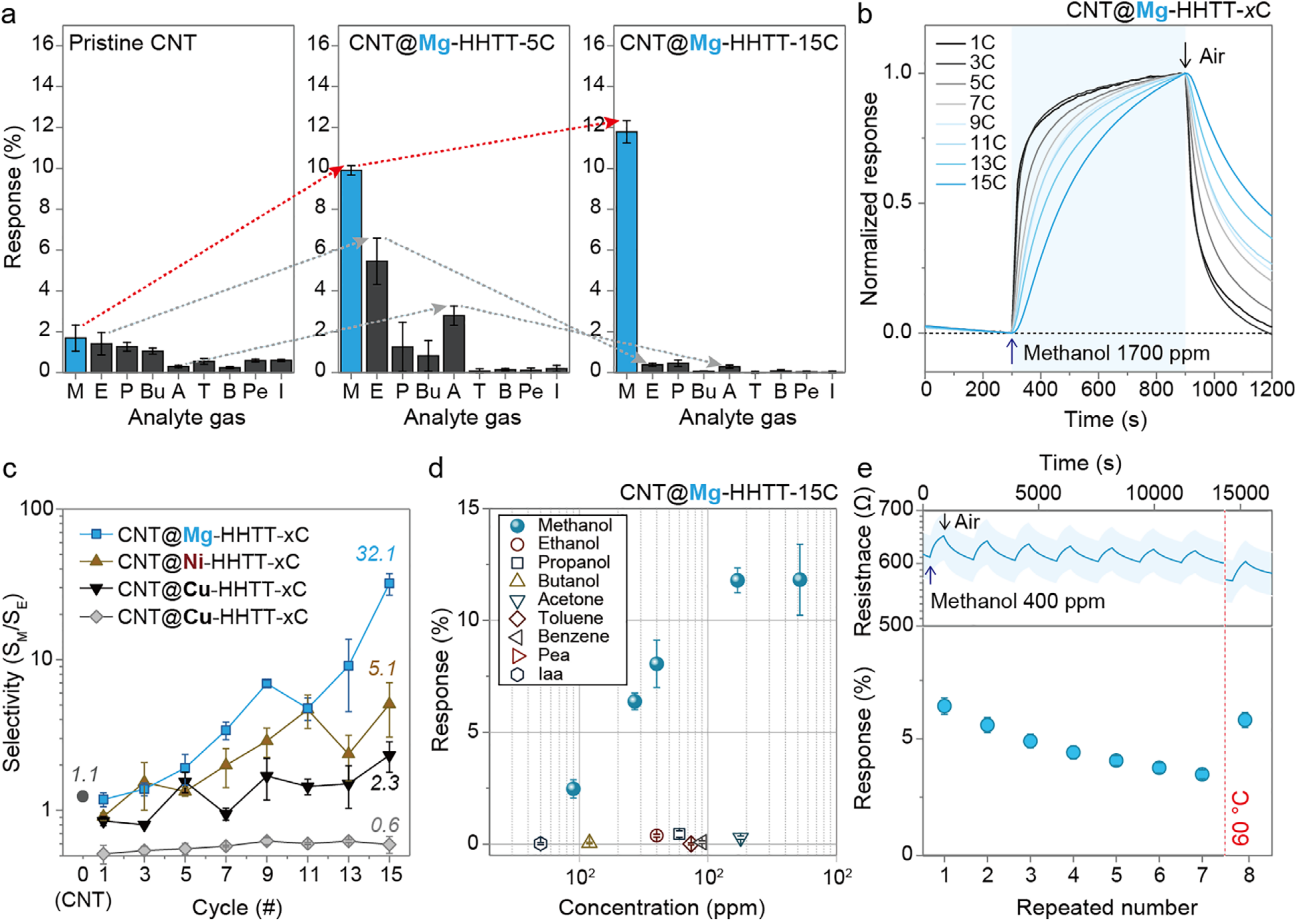
(a) Gas responses of CNT, CNT@Mg‐HHTT‐5C, and CNT@Mg‐HHTT‐15C sensors to methanol (M), ethanol (E), propanol (P), butanol (Bu), acetone (A), toluene (T), benzene (B), phenethyl alcohol (Pe), and isoamyl alcohol (I). (b) Normalized response profiles of CNT@Mg‐HHTT‐*x*C sensors. (c) Methanol‐to‐ethanol selectivity of CNT@M‐HHTT‐*x*C sensors (M = Cu, Ni, Mg). (d) Responses of CNT@Mg‐HHTT‐15C sensors to methanol concentrations ranging from 90 to 5200 ppm and to representative interfering gases at room temperature. (e) Repetitive sensing response of CNT@Mg‐HHTT‐15C sensors to 400 ppm methanol. Error bars in all panels represent the standard deviation calculated from measurements of three independent sensors.

Beyond five coating cycles, the methanol responses of all sensors approach saturation, with only marginal additional increases observed (Figure 3a). This behavior indicates that further growth of cMOF layers beyond this thickness does not substantially increase the number of sites that effectively couple to the conductive CNT network. This trend is illustrated for CNT@Mg‐HHTT‐xC sensors in Figure 4a, where the methanol response increases from 9.97% at five cycles to 11.79% after fifteen cycles. In contrast, responses to other VOCs exhibit a markedly different dependence on coating thickness. For example, ethanol and acetone responses increase from 1.69% for pristine CNTs to 5.44% and 2.75%, respectively, after five Mg‐HHTT coating cycles, but decrease to 0.38% and 0.29% at fifteen cycles. Responses of CNT@Mg‐HHTT‐15C sensors to additional interferents, including propanol, butanol, toluene, benzene, phenylethanol, and isoamyl alcohol, are likewise suppressed to negligible levels (< 0.47%).

This evolution in response behavior indicates that Mg‐HHTT layers deposited beyond five cycles function primarily as size‐selective filtering layers rather than as catalytic amplification layers. Consistent with this interpretation, normalized transient responses for methanol (Figure 4b) show a systematic decrease in response rate with increasing Mg‐HHTT thickness, reflecting restricted gas transport through thicker coatings. Methanol, with a kinetic diameter of 3.6 Å, retains sufficient permeability through the Mg‐HHTT layers, whereas larger molecules such as ethanol (4.5 Å) and acetone (4.6 Å) experience significantly greater diffusion resistance. As a result, Mg‐HHTT coatings preferentially transmit methanol while attenuating transport of larger interferents. Cu‐HHTT and Ni‐HHTT coatings exhibit qualitatively similar trends, although the magnitude of the filtering effect is substantially smaller than that observed for Mg‐HHTT (Figures S18–S20).

Figure 4c summarizes the methanol‐to‐ethanol selectivity (S_M_/S_E_) of CNT@M‐HHTT‐*x*C (M = Cu, Ni, Mg) and CNT@Cu‐HHTP‐xC sensors as a function of coating thickness (*x* = 1–15). For CNT@Mg‐HHTT‐*x*C sensors, selectivity increases monotonically with coating number and reaches 32.1 at *x* = 15, substantially higher than that of pristine CNTs (S_M_/S_E_ = 1.1). In contrast, Cu‐HHTT and Cu‐HHTP coatings do not produce a comparable enhancement in selectivity. Even at high thickness, CNT@Cu‐HHTT‐15C and CNT@Cu‐HHTP‐15C exhibit low selectivity values of 2.3 and 0.6, respectively. These results indicate that the high methanol selectivity of Mg‐HHTT‐coated CNT sensors arises from the presence of intra‐pore clusters that restrict pore accessibility. Consistent with this interpretation, CNT@Ni‐HHTT‐15C sensors containing dispersed IPCs display an intermediate selectivity of 5.1, falling between CNT@Mg‐HHTT‐15C (S_M_/S_E_ = 32.1) and CNT@Cu‐HHTT‐15C (S_M_/S_E_ = 2.3).

The response of CNT@Mg‐HHTT‐15C sensors to methanol concentrations ranging from 90 to 5200 ppm was evaluated at room temperature (Figure 4d). Across this range, methanol is detected selectively, with negligible responses to larger interfering species. By comparison, CNT@Ni‐HHTT‐15C and CNT@Cu‐HHTT‐15C sensors show greater susceptibility to interference, limiting their selectivity relative to CNT@Mg‐HHTT‐15C (Figure S21). Sensor repeatability was assessed using repeated exposures to 400 ppm methanol (Figure 4e). After seven exposure cycles, a modest decrease in sensitivity is observed, consistent with slower desorption and diffusion associated with thicker cMOF layers. Sensitivity is recovered following mild heating at 60°C for 5 min, indicating that methanol sensing in these devices is reversible.

### 2.3 Molecular Dynamics Simulations of cMOFs with Methanol and Ethanol

**FIGURE 5:**
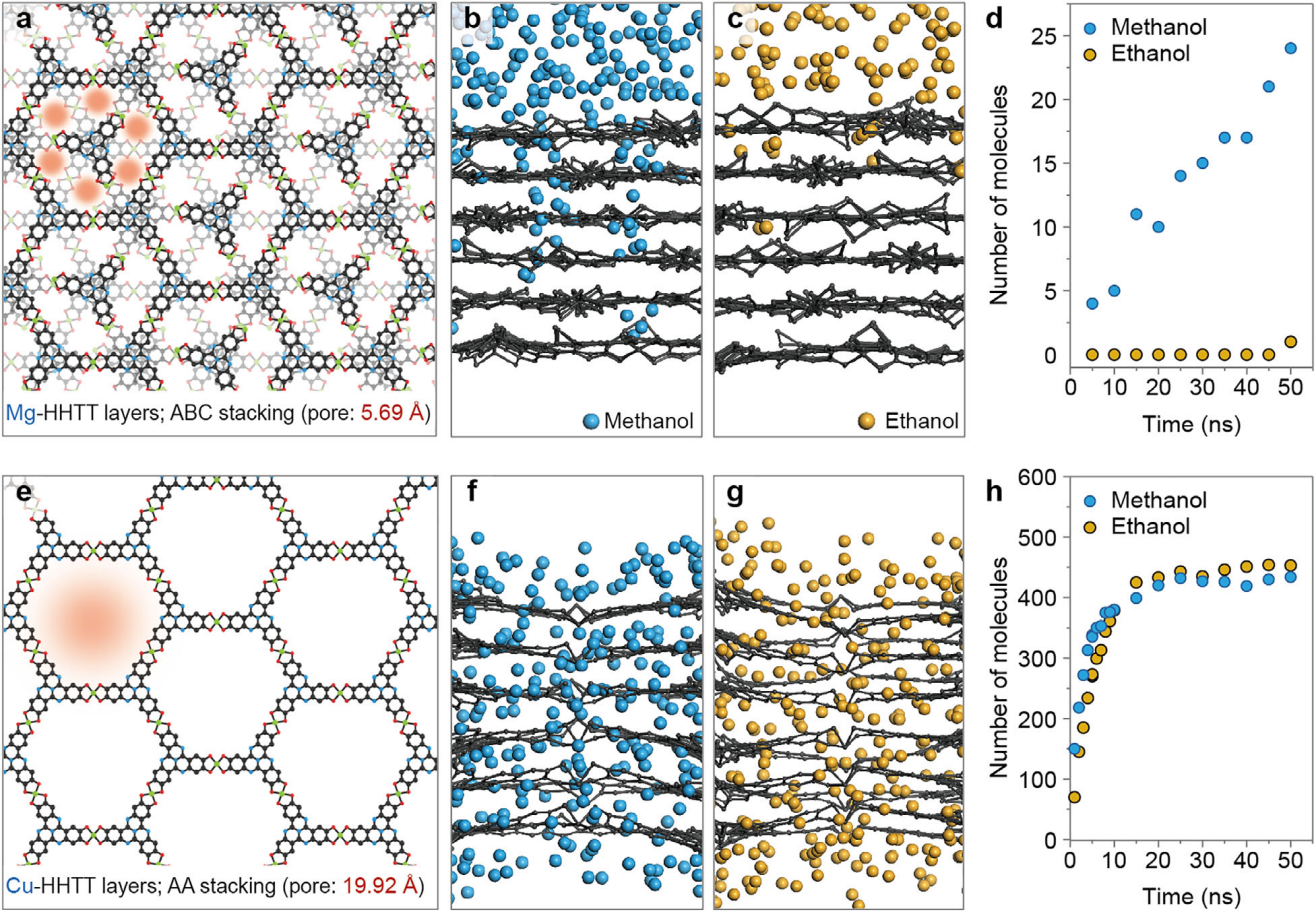
Structural representations of (a) Mg‐HHTT and (e) Cu‐HHTT. Molecular dynamics simulation snapshots showing methanol (blue) and ethanol (yellow) permeation through (b,c) Mg‐HHTT and (f,g) Cu‐HHTT. Time‐dependent counts of permeating molecules for (d) Mg‐HHTT and (h) Cu‐HHTT. The permeation of molecules through the MOF was deemed successful when the oxygen atoms in methanol and ethanol seamlessly passed the third layer of the MOF structure.

After a 50 ns MD simulation, clear differences in permeation behavior are observed between Mg‐HHTT and Cu‐HHTT. In Mg‐HHTT, methanol and ethanol permeation differs substantially, yielding an N_M_/N_E_ ratio of 24 (Figure 5b–d). In contrast, Cu‐HHTT exhibits nearly equal permeation with an N_M_/N_E_ ratio of 0.96 (Figure 5f–h). This difference in selectivity arises from the presence or absence of IPC‐mediated pore confinement. Pore size analysis during the simulations yields an average pore diameter of 5.69 Å for Mg‐HHTT and 19.92 Å for Cu‐HHTT (Figure 5a,e, and Figure S22). The smaller pores in Mg‐HHTT impose stronger constraints on ethanol diffusion than on methanol diffusion, thereby limiting ethanol access to the CNT surface. In contrast, the larger pores in Cu‐HHTT permit both molecules to diffuse with minimal restriction, reducing its effectiveness as a molecular sieving layer. To probe this mechanism under more realistic structural conditions, we performed additional MD simulations considering IPC defects and stacking disorder that may arise during LBL film growth. Even when moderate IPC defects and non‐ideal stacking configurations were introduced, both methanol and ethanol permeation increased due to enlarged pore accessibility, but ethanol transport remained significantly more restricted than methanol (Figures S23 and S24). These results indicate that the methanol selective transport behavior of Mg‐HHTT is preserved even in the presence of structural imperfections. Taken together, the MD and DFT results identify Mg‐HHTT as the most effective framework among those examined for selective methanol sensing.

The mechanism underlying selective methanol and ethanol detection was examined using MD simulations. Structural models of Mg‐HHTT and Cu‐HHTT were constructed based on experimentally validated frameworks [22]. Because stacking order strongly influences host–guest interactions and gas permeation in two dimensional cMOFs [26, 27], distinct stacking motifs were employed for each system. Mg‐HHTT was modeled using a twisted‐staggered ABC stacking arrangement incorporating IPCs, whereas Cu‐HHTT was represented using an eclipsed AA stacking configuration without IPCs. For each model, 1000 methanol and ethanol molecules were placed with a minimum separation tolerance of 2.5 Å to avoid initial overlap [28], and the numbers of methanol (N_M_) and ethanol (N_E_) molecules permeating through the MOF layers were quantified.

### 2.4 Detection of Methanol in Liquors Under Practical Conditions

**FIGURE 6:**
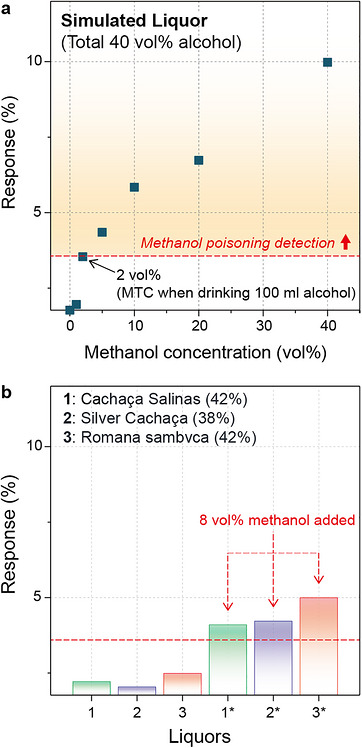
(a) Responses of CNT@Mg‐HHTT‐15C sensors as a function of methanol content in methanol‐spiked liquor containing 40 vol% alcohol. The red line denotes the maximum tolerance concentration (MTC) of methanol [29] for human consumption of 100 mL of alcoholic beverage. (b) Detection of methanol content in selected commercial liquors (1: Cachaça Salinas, 2: Silver Cachaça, 3: Romana Sambvca) and in methanol‐tainted liquors (asterisk indicates addition of 8 vol% methanol). The experiment was conducted under ambient humidity conditions.

The applicability of CNT@Mg‐HHTT‐15C sensors for detecting methanol contamination in alcoholic beverages was evaluated by calibrating the sensors with a prepared liquor sample containing a fixed total alcohol content of 40%, in which ethanol was partially replaced by methanol at concentrations of 1%, 2%, 5%, 10%, 20%, and 40% (Figure 6a). During calibration, sensors were stabilized in ambient air at room temperature, exposed for 2 min to vapor from the headspace of a container holding 20 mL of sample liquor, and subsequently allowed to recover in ambient air (Figure S25). Pure ethanol samples produced a low sensor response of 1.50%, whereas sensor responses increased systematically with increasing methanol content. Given that the maximum tolerance concentration (MTC) of methanol is 2 vol% for consumption of 100 mL of 40% alcohol [29], CNT@Mg‐HHTT‐15C sensor responses exceeding 3.05% indicate potentially hazardous contamination levels. In addition, to confirm the long‐term stability of the CNT@Mg‐HHTT‐15C sensor under ambient humid conditions, the resistance and sensing responses toward methanol and ethanol were investigated (Figure S26). The resistance remained nearly constant over 21 days, indicating that the CNT‐based conduction pathway is stable. In terms of sensing characteristics, excluding periods of elevated indoor humidity (e.g., rainy conditions), the sensor maintained reversible selectivity toward methanol over 3 weeks. If the humidity dependence under high‐humidity conditions can be mitigated, our CNT@cMOF sensor can be used very stably.

Using this calibration, three commercial spirits—Cachaça Salinas (42%), Silver Cachaça (38%), and Romana sambvca (42%)—were examined (Figure 6b). Exposure of the sensors to these beverages for 2 min resulted in responses of 1.82%, 1.60%, and 1.88%, respectively, consistent with safe methanol levels. Despite the presence of strong aromatic components, interference effects were negligible. In contrast, deliberate addition of 8 vol% methanol to each beverage produced substantially higher sensor responses indicative of contamination risk. Selective sensing in mixed methanol–ethanol systems was further supported by MD simulations (Figure S27). Simulations incorporating equal numbers of methanol and ethanol molecules within the Mg‐HHTT framework yield a permeation ratio of N_M_/N_E_ = 22, confirming preferential methanol transport. Collectively, these results demonstrate that CNT@Mg‐HHTT‐15C sensors enable rapid detection of methanol in alcoholic beverages even in the presence of ethanol and aromatic interferents.

## 3 Conclusion

In this work, we have successfully designed methanol sensors employing Mg‐HHTT functionalized CNT chemiresistors that operate effectively at room temperature. The enhancement in sensitivity is attributed to the formation of abundant gas reaction sites facilitated by the unique structure of Mg‐HHTT, which includes metal clusters, nitrogen sites of the ligand, and additional IPCs configurations. Moreover, the Mg‐HHTT coating layer exhibits exceptional selectivity for methanol over ethanol, a result of the pore confinement effects of IPCs and the twisted ABC stacking modes. This gas‐sensing mechanism has been substantiated through DFT and MD simulations. The practicality of our approach is demonstrated by the tests with methanol‐tainted alcoholic beverages, confirming its potential for real‐world applications.

## 4 Experimental Section/Methods

### 4.1 Materials

5,6‐Dimethoxyanthranil (CAS 148495‐00‐5; 95%; Synnovator Inc.), 2,3,6,7,10,11‐hexahydroxytriphenylene (CAS 4877‐80‐9; 95%; 95%; TCI chemical), ammonium acetate (CAS 631‐61‐8; 98%; Sigma–Aldrich), acetic acid (CAS 64‐19‐8; 99%; Sigma–Aldrich), sulfolane (CAS 126‐33‐0; 99%; Sigma–Aldrich), pyridine hydrochloride (CAS 628‐13‐7; 98%; Sigma–Aldrich), CuSO_4_·5H_2_O (CAS 758‐99‐8; 99%; Alfa Aesar), Ni(OAc)_2_·4H_2_O (CAS 6018‐89‐9; 98%; Thermoscientific), Mg(OAc)_2_·4H_2_O (CAS 16674‐78‐5; 99%; Strem Chemicals), ethanol (CAS 64‐17‐5; KOPTEC). All reagents and solvents were used without further purification.

### 4.2 Synthesis of 2,3,7,8,12,13‐Hexahydroxytricycloquinazoline (HHTT) Ligand

Conditions for the synthesis of H_6_HHTT were adapted from a previous report [22]. A mixture of acetic acid (15 mL) and sulfolane (75 mL) containing 5,6‐dimethoxyanthranil (4.28 g) and ammonium acetate (12.3 g) was charged into a Schlenk flask and heated under reflux for 96 h. The resulting dark brown hexamethoxytricycloquinazoline was isolated by centrifugation and washed with deionized water and methanol. Hexamethoxytricycloquinazoline (0.810 g) was then combined with pyridine hydrochloride (27.4 g) and heated under reflux for 3 h. After cooling to room temperature, the resulting HHTT ligand was collected by centrifugation and washed with deionized water.

### 4.3 Preparation of CNT@M‐HHTT Sensors (M = Cu, Ni, Mg)

Carboxylic acid–functionalized single‐walled carbon nanotubes (CNTs) were dispersed in DMF at a concentration of 0.05 mg/mL and sonicated for 1 h to ensure uniform dispersion and minimize aggregation. An aliquot (5 µL) of the resulting suspension was drop‐cast onto Au interdigitated alumina substrates to form the CNT sensing layer. M‐HHTT (M = Cu, Ni, Mg) coatings were deposited onto the CNTs using a layer‐by‐layer method. Metal precursor solutions consisted of 1 mm ethanol solutions of Mg(II) acetate tetrahydrate, Ni(II) acetate tetrahydrate, or Cu(II) sulfate pentahydrate, while the ligand solution comprised a 0.1 mm ethanol solution of H_6_HHTT. In each deposition cycle, the CNT‐coated substrates were immersed sequentially in the metal solution for 2 min and the ligand solution for 4 min. After each immersion step, the substrates were rinsed with ethanol to remove residual metal and ligand salts.

### 4.4 Synthesis of M_3_HHTT_2_ (M = Cu, Ni) and M_6_HHTT_3_ (M = Ni, Mg)

The synthesis of M_3_HHTT_2_ (M = Cu, Ni) and M_6_HHTT_3_ (M = Ni, Mg) was carried out following previously reported procedures [22]. Cu_3_HHTT_2_ was prepared by adding 2.1 mL of an aqueous solution containing 45 mg of CuSO_4_·5H_2_O to 0.9 mL of a DMF solution containing 50 mg of H_6_HHTT, followed by heating at 85°C for 12 h. Ni_3_HHTT_2_ was synthesized by adding 0.8 mL of an aqueous solution containing 15 mg of Ni(OAc)_2_·4H_2_O to 0.2 mL of a DMF solution containing 10 mg of H_6_HHTT and heating at 85°C for 12 h. Ni_6_HHTT_3_ was obtained by adding 2 mL of an aqueous solution containing 12 mg of Ni(OAc)_2_·4H_2_O to 10 mL of an aqueous solution containing 50 mg of H_6_HHTT, followed by heating at 85°C for 12 h. Mg_3_HHTT_2_ was prepared by adding 0.9 mL of an aqueous solution containing 16.5 mg of Mg(OAc)_2_·4H_2_O to 0.4 mL of a DMF solution containing 16 mg of H_6_HHTT and heating at 85°C for 12 h.

### 4.5 Laboratory Powder X‐Ray Diffraction

PXRD patterns were acquired in reflection mode using a Bruker Advance II diffractometer equipped with Ni‐filtered Cu Kα radiation (Kα_1_ = 1.5406 Å, Kα_2_ = 1.5444 Å, ratio Kα_2_/Kα_1_ = 0.5). The sample, thinly spread on a zero‐background silicon substrate, was scanned at a rate of 2°/min to collect the patterns.

### 4.6 Scanning Electron Microscopy

SEM analysis was conducted at MIT.nano using the Zeiss Gemini 450 high‐resolution scanning electron microscope, utilizing InLens or SE2 detection, and operated at a voltage of 5.00 kV.

### 4.7 Transmission Electron Microscopy

TEM was performed at MIT MRSEC (Center for Materials Science and Engineering) on a FEI Tecnai 120 kV electron microscope.

### 4.8 X‐Ray Photoelectron Spectroscopy

XPS was performed at the MIT MRSEC (formerly the Center for Materials Science and Engineering) using a Physical Electronics PHI Versaprobe II X‐ray photoelectron spectrometer installed with an Al anode.

### 4.9 Raman Spectroscopy

Raman spectroscopy at MIT MRSEC (Center for Materials Science and Engineering) on a Renishaw Invia Reflex Raman Confocal Microscope.

### 4.10 Gas Sensing Experiments

Gas sensing measurements were performed using a home‐built gas flow system integrated with a sensor array chamber capable of accommodating up to 16 sensors and equipped with inlet and outlet ports for controlled gas delivery. In this setup, one mass flow controller (MFC) was connected to an N‐TEK FlexStream gas generator (Kin‐Tek Laboratories, La Marque, TX) to produce VOCs, including methanol, ethanol, propanol, butanol, acetone, toluene, benzene, phenylethanol, and isoamyl alcohol, while a second MFC supplied dry air to stabilize the sensors (Figure S13). The total flow rate was maintained at 1000 sccm. Prior to room‐temperature sensing measurements, the sensors were stabilized and activated at 75 °C for 30 min. Resistance transients were recorded using a DAQ970A multimeter (Keysight) interfaced with a personal computer.

### 4.11 Methanol‐Tainted Liquor Tests

For practical evaluation, we used a static chamber system that contained both the sensors and the liquor samples. Tested liquors included commercial alcoholic beverages (Cachaça Salinas (42%), Silver Cachaça (38%), and Romana Sambuca (42%)) as well as liquors deliberately contaminated with methanol in concentrations ranging from 0 to 40 vol%. Long‐term sensor stability tests were conducted under ambient humid conditions over 3 weeks. Prior to each measurement, the sensors were pre‐treated in an oven at 60 °C for 5 min, and their responses to 40% methanol and ethanol were compared within the chamber.

### 4.12 MD Simulation Model Preparation

Monolayer models of Mg‐HHTT and Cu‐HHTT, the latter lacking an IPC, were constructed as the basis for MD simulations. These models were subsequently optimized using DFT to obtain relaxed unit cells and atomic positions consistent with experimental structures. To model methanol and ethanol permeation, six‐layer assemblies were generated using twisted‐staggered ABC stacking for Mg‐HHTT and eclipsed AA stacking for Cu‐HHTT. The simulation cell incorporated extended vacuum regions above and below the MOF layers (perpendicular to the framework plane) to permit unrestricted molecular motion. Three simulation conditions were considered: systems containing 1000 methanol molecules, 1000 ethanol molecules, and an equimolar mixture of 500 methanol and 500 ethanol molecules. Periodic boundary conditions in the z‐direction were constrained by potential walls positioned at z = 0 and 80 Å, ensuring that molecular permeation occurred exclusively through the MOF layers.

### 4.13 MD Simulation Details

MD simulations were executed utilizing the Large‐scale atomic/molecular massively parallel simulator (LAMMPS) software, operating under the NVT ensemble [30]. The universal force field (UFF) and the charge equilibration method (QEq) were diligently applied across all atoms [31, 32]. We ensured flexibility in the molecules and MOF by applying various intra‐molecular force field parameters like harmonic bond, and a hybrid style of cosine/periodic and fourier angle, alongside harmonic dihedral and fourier improper [33]. The inter‐molecular interaction between atoms was deduced by combining the 12–6 Lennard‐Jones and Coulombic potentials, with cutoff values of 12.5 and 6 Å, respectively. The operational parameters of the simulation were set at a time span of 50 ns, with a 1 fs time step, and conducted at conditions of 298 K and 1 bar. The permeation of molecules through the MOF was deemed successful when the oxygen atoms in methanol and ethanol seamlessly passed the third layer of the MOF structure. For the pore size distribution of Mg‐HHTT and Cu‐HHTT, the MD snapshots were extracted over a period of 100 ps, and the pore sizes of each structure were precisely calculated utilizing the Zeo++ module [34].

### 4.14 DFT Simulation Details

The generalized gradient approximation, along with the Perdew‐Burke‐Ernzerhof functional, was utilized for exchange correlation interactions [35]. The projector augmented‐wave method was also incorporated to enhance accuracy. For improved representation of van der Waals dispersion between methanol and MOF, Grimme's D3 method was applied [36, 37]. A cut‐off energy of 520 eV was designated for the plane wave basis set. An electronic and ionic relaxation criterion was established as 10^−5^ eV and 0.003 eV/Å, respectively. The Gaussian smearing scheme was implemented with a sigma value of 0.05 eV. To obtain a more precise electronic structure of transition metals, spin polarization was considered. For the geometrical optimization of MOFs and methanol binding energy calculations, k‐points were systematically arranged in a gamma‐centered 2 × 2 × 3 grid and a 1 × 1 × 1 grid within the Brillouin zone, respectively. The binding energies between a methanol molecule and MOF were calculated via the following equation, *E_binding_* = *E*_*MOF* + methanol_  − *E_MOF_* − *E_methanol_*, where *E_binding_*, *E*_*MOF* + methanol_, *E_MOF_* and *E_methanol_* are binding energy, total energy of MOF containing methanol molecule, the total energy of pure MOF, and the total energy of the methanol molecule, respectively. To identify the preferred adsorption configuration of methanol at the metal, linker, and IPC sites, we systematically examined several possible starting geometries. In each case, methanol was initially placed at chemically reasonable adsorption sites, and the structures were fully relaxed to determine the most stable configuration. The binding configurations used to determine the linker adsorption sites are presented in Figures S28 and S29. Water molecules bound to the IPC clusters were considered during the methanol‐IPC binding energy calculation [22]. All calculations were conducted via the Vienna Ab initio Simulation Package (VASP) version 5.4.1 [38, 39].

## Conflicts of Interest

The authors declare no conflicts of interest.

## Supporting information




**Supporting File**: adma73406‐sup‐0001‐SuppMat.pdf.

## Data Availability

The data that supports the findings of this study are available in the supplementary material of this article.
