# Supplementary material for: Methanol‐Ethanol Discrimination and Selective Sensing Enabled by Molecular Sieving in Conductive MOFs
Source: Adv Mater. 2026 May 19;38(35):e73406. doi: 10.1002/adma.73406 (PMC13288196; doi:10.1002/adma.73406)
Supplement: Supplementary file 1 — Supporting File: adma73406‐sup‐0001‐SuppMat.pdf. [file ADMA-38-e73406-s001.pdf]

Supporting Information

Methanol-Ethanol Discrimination and Selective Sensing Enabled by Molecular Sieving in Conductive MOFs

*Young-Moo Jo†, Mingyu Jeon†, Dong-Ha Kim, Jihan Kim, and Mircea Dincă**

Y.-M. Jo, M. Jeon, D.-H. Kim, M. Dincă

Department of Chemistry, Massachusetts Institute of Technology, Cambridge, Massachusetts 02139, USA

M. Jeon, J. Kim

Department of Chemical and Biomolecular Engineering, Korea Advanced Institute of Science and Technology, Daejeon 34141, Republic of Korea

Y.-M. Jo

School of Materials Science and Engineering, Kyungpook National University, Daegu 41566, Republic of Korea

D.-H. Kim

Present address: Department of Materials Science and Chemical Engineering, Hanyang University, Ansan 15588, Republic of Korea

M. Dincă

Department of Chemistry, Princeton University, Princeton, New Jersey 08540, USA

† These authors contributed equally.

*To whom correspondence should be addressed to mdinca@princeton.edu

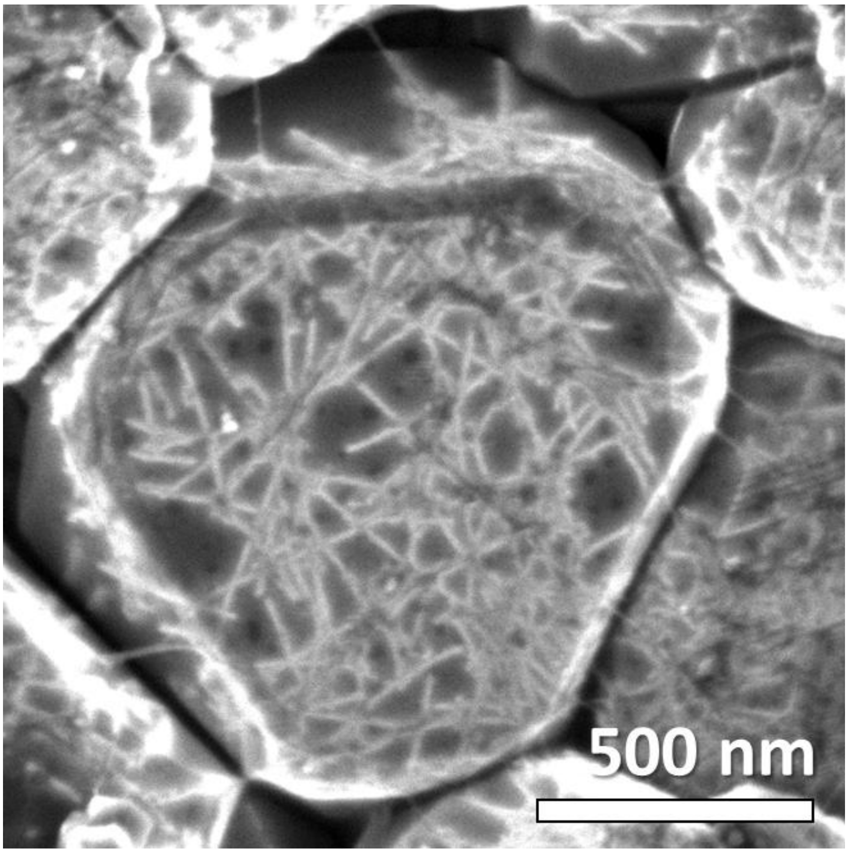

Figure S1. SEM image of percolated pristine single-walled CNT.

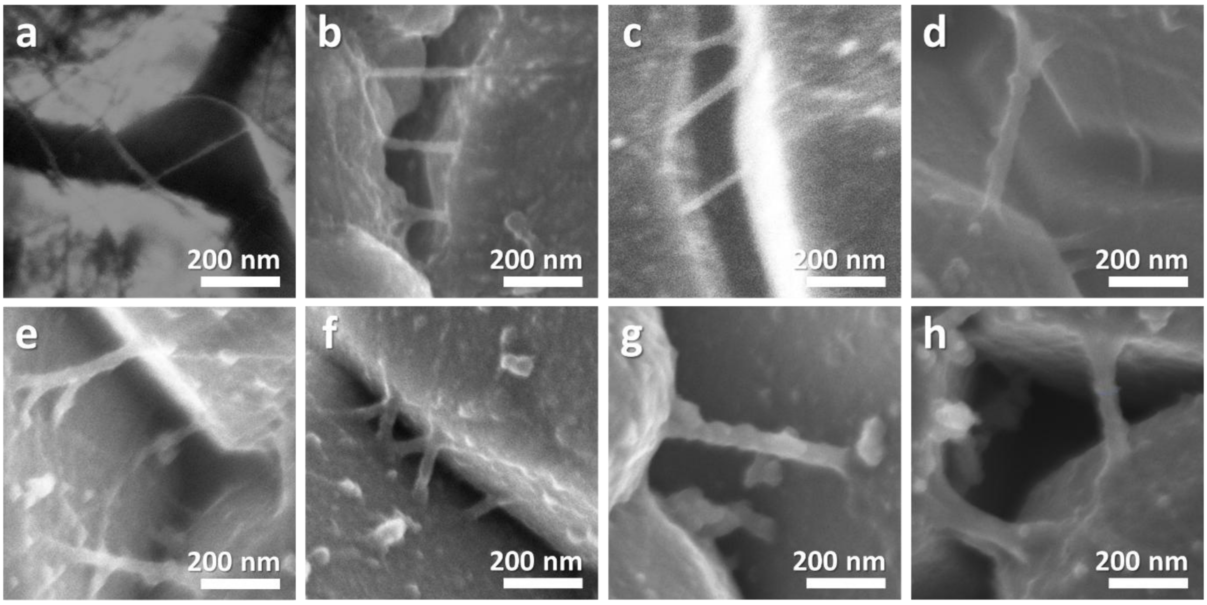

Figure S2. SEM images of CNT@Mg-HHTT-*x*C (*x* = 1, 3, 5, 7, 9, 11, 13, and 15). To clearly observe the growth of cMOF on the CNT surface, suspended CNTs located between alumina substrate grains were analyzed.

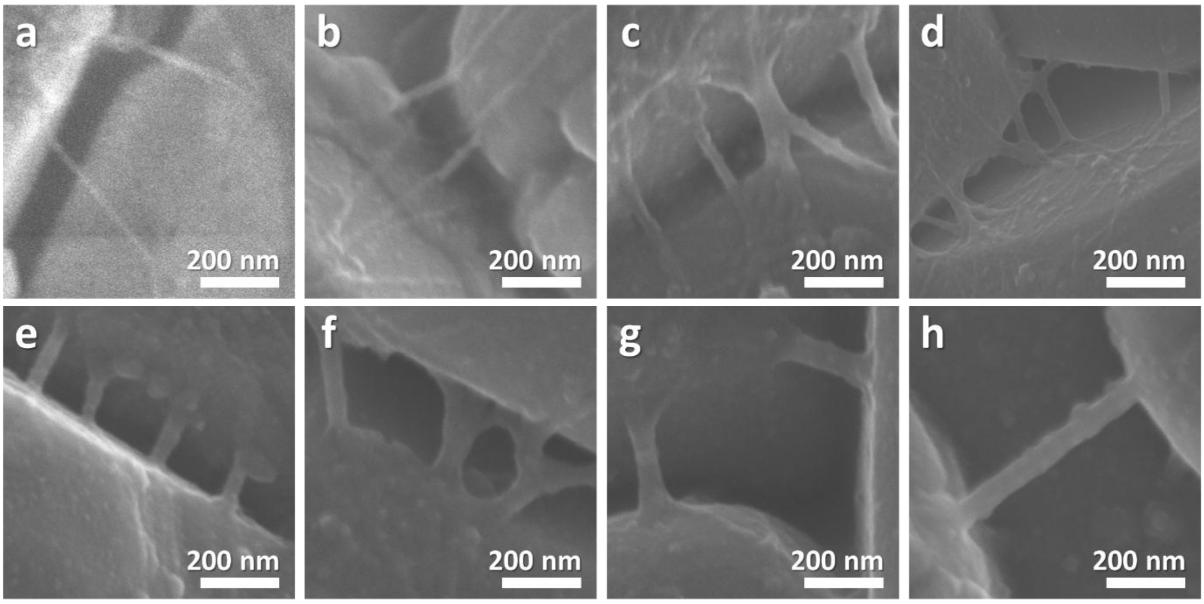

Figure S3. SEM images of CNT@Ni-HHTT-*x*C (*x* = 1, 3, 5, 7, 9, 11, 13, and 15). To clearly observe the growth of cMOF on the CNT surface, suspended CNTs located between alumina substrate grains were analyzed.

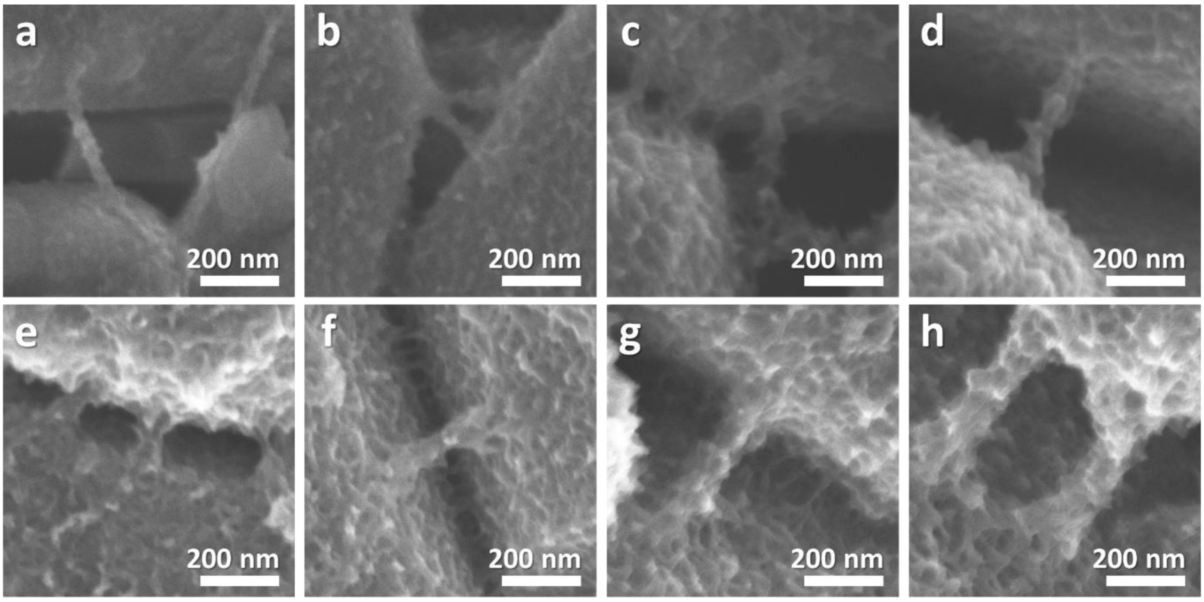

Figure S4. SEM images of CNT@Cu-HHTT-*x*C (x = 1, 3, 5, 7, 9, 11, 13, and 15). To clearly observe the growth of cMOF on the CNT surface, suspended CNTs located between alumina substrate grains were analyzed.

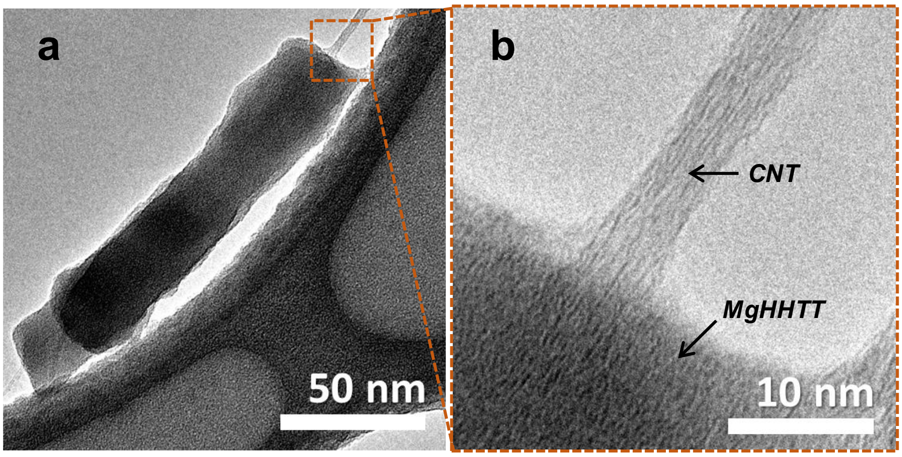

Figure S5. (a) TEM images of CNT@Mg-HHTT-15C sensors. (b) Inset image of (a).

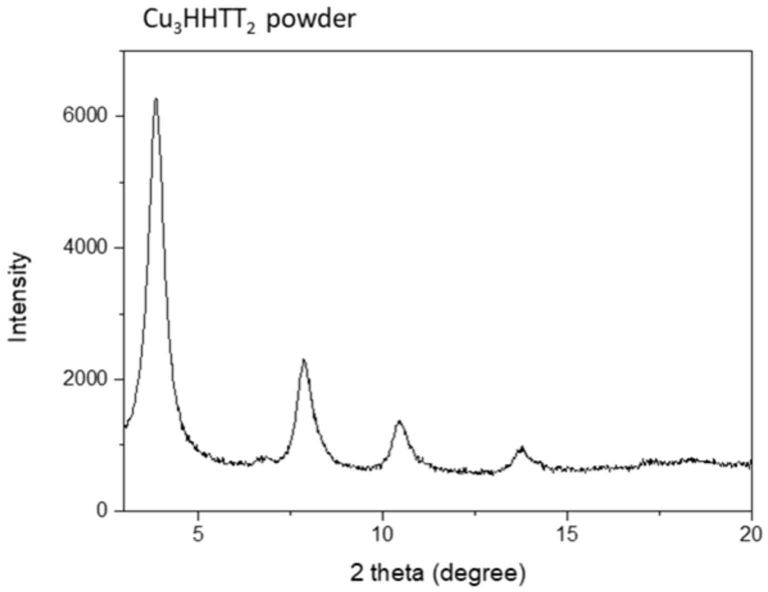

Figure S6. Laboratory PXRD patterns for Cu_3HHTT_2 powders, consistent with the reported pattern in the literature.[S1]

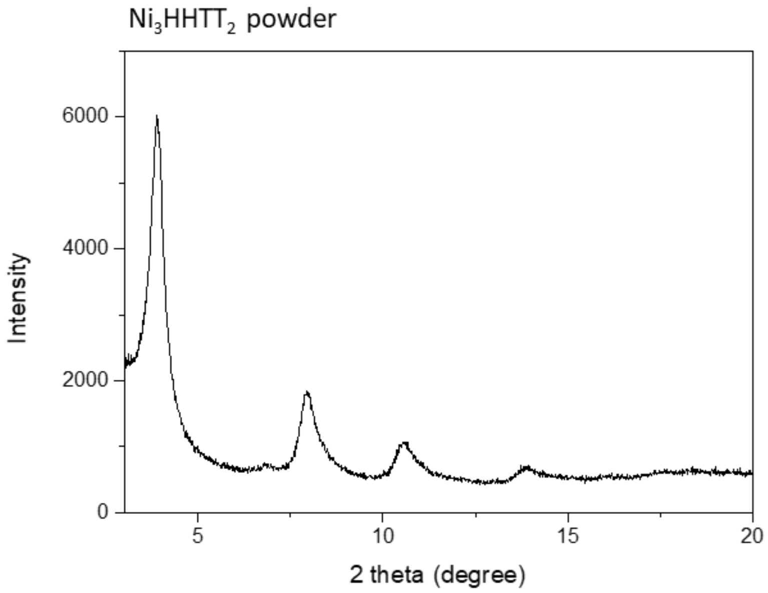

Figure S7. Laboratory PXRD patterns for Ni_3HHTT_2 powders, consistent with the reported pattern in the literature.[S1]

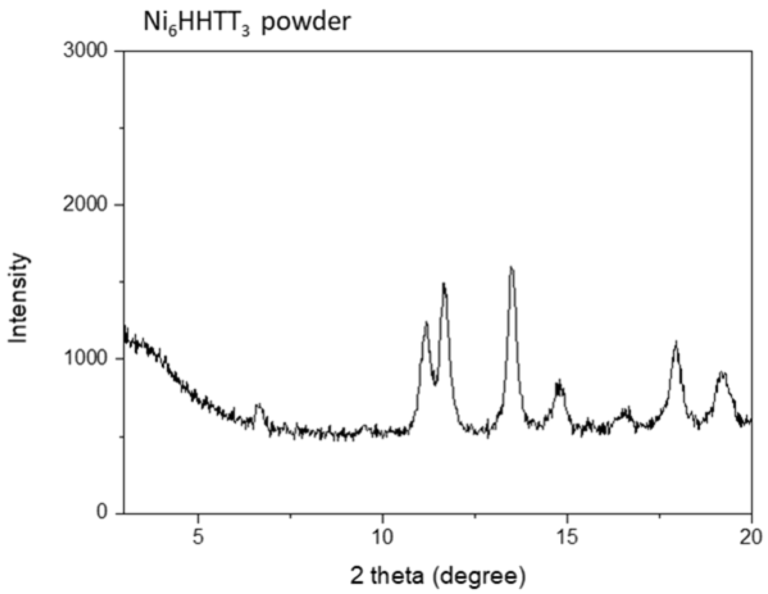

Figure S8. Laboratory PXRD patterns for Ni_6HHTT_3 powders, consistent with the reported pattern in the literature.[S1]

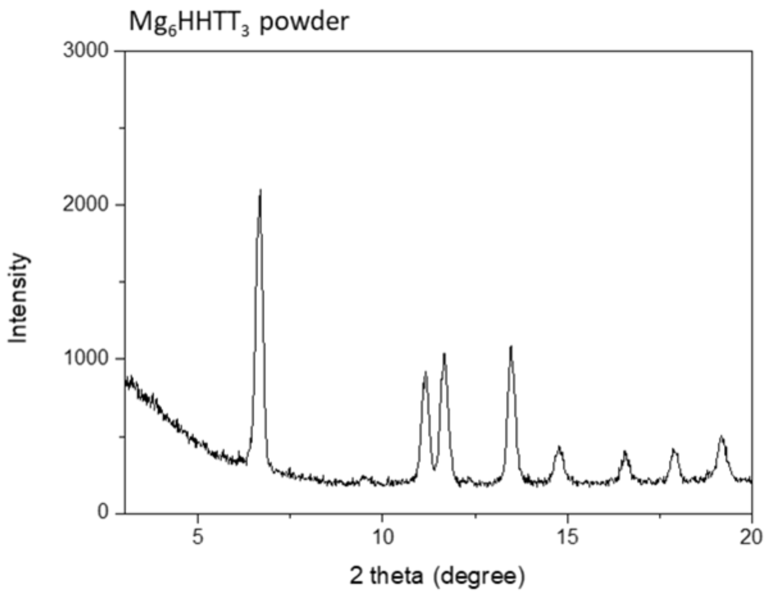

Figure S9. Laboratory PXRD patterns for Mg_6HHTT_3 powders, consistent with the reported pattern in the literature.[S1]

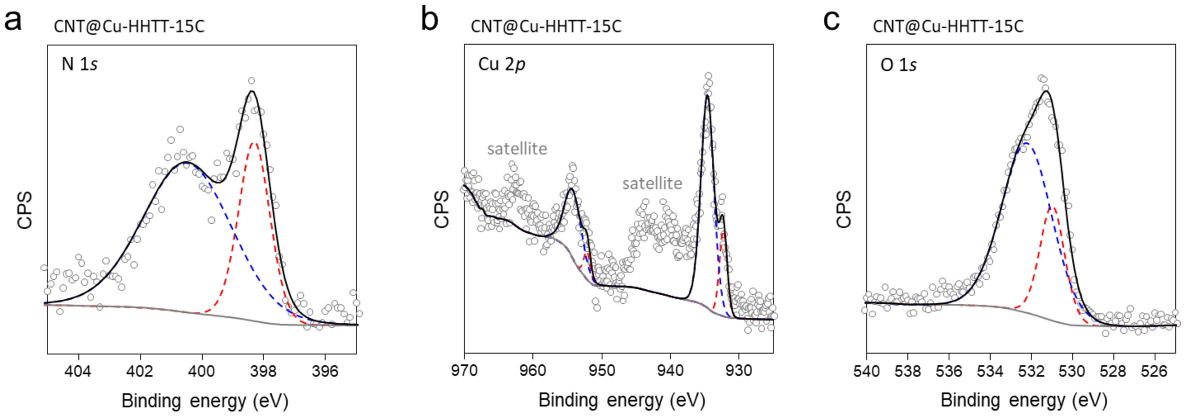

Figure S10. XPS analysis of (a) N 1*s*, (b) Cu 2*p*, and (c) O 1s for CNT@Cu-HHTT-15C.

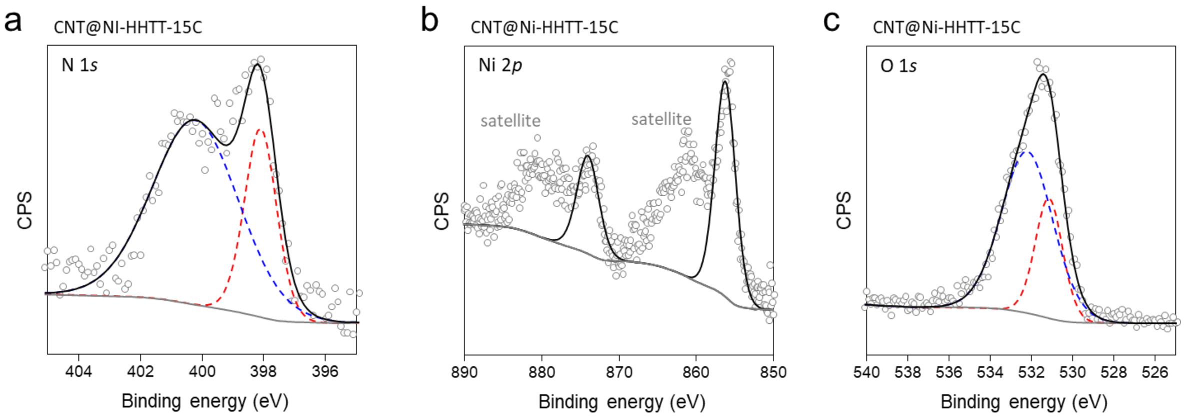

Figure S11. XPS analysis of (a) N 1*s*, (b) Ni 2*p*, and (c) O 1s for CNT@Ni-HHTT-15C.

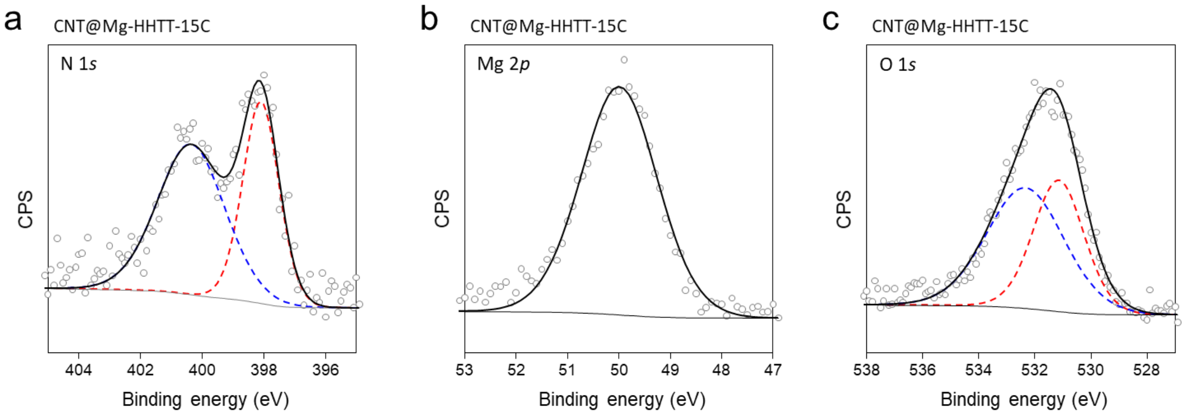

Figure S12. XPS analysis of (a) N 1*s*, (b) Mg 2*p*, and (c) O 1s for CNT@Mg-HHTT-15C.

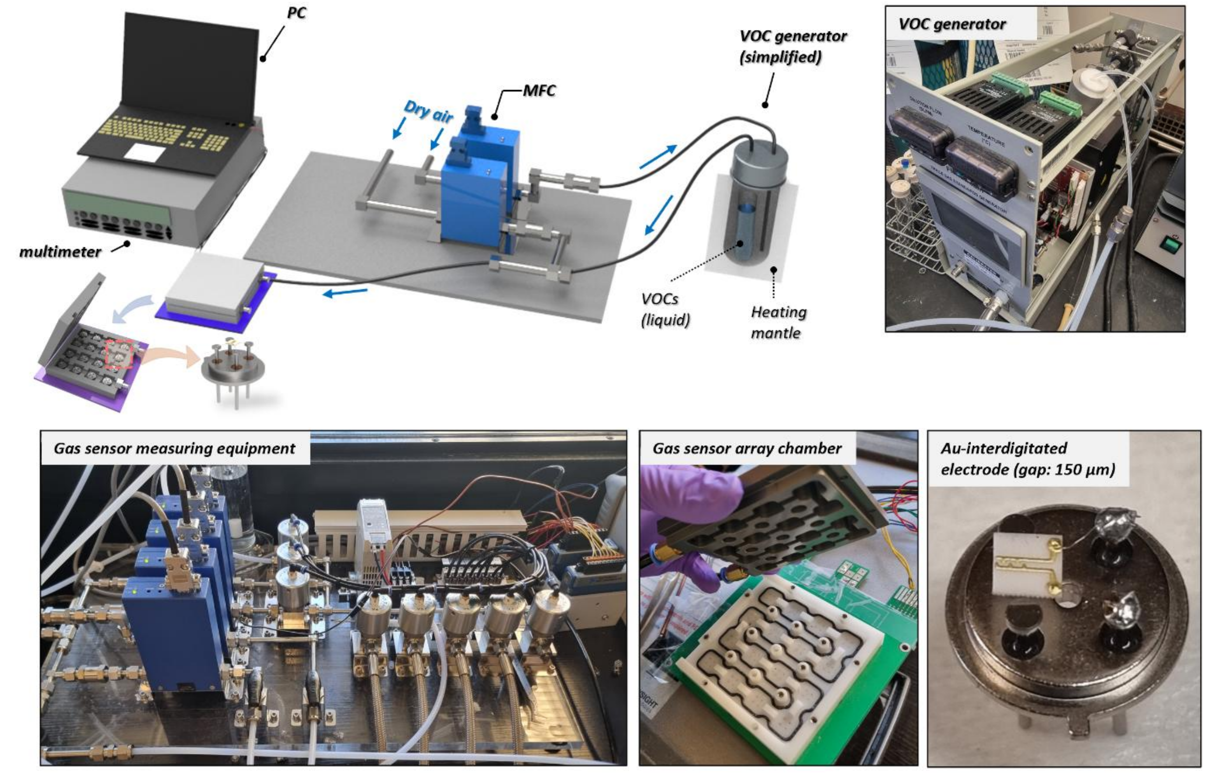

Figure S13. Simplified illustrations and photos show a home-made gas flow system equipped with a VOC generator and a gas sensing array chamber. The VOC generators are set to 40 °C for all VOC solvents, so the concentrations of gases vary depending on their evaporation rates: 1700 ppm for methanol, 400 ppm for ethanol, 600 ppm for propanol, 120 ppm for butanol, 1800 ppm for acetone, 900 ppm for benzene, 50 ppm for isoamyl alcohol, and 4 ppm for phenethyl alcohol. For methanol, various temperatures were used to generate different concentrations (90, 270, 400, 1700, and 5200 ppm).

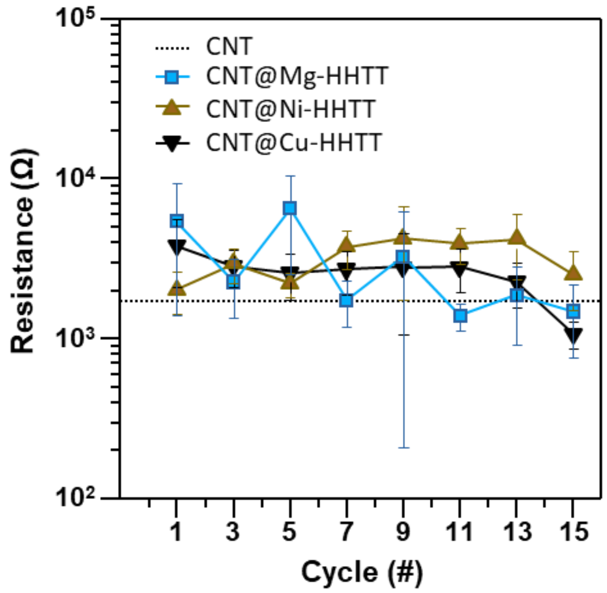

Figure S14. Resistance of CNT and CNT@M-HHTT-xC (M=Cu, Ni, Mg; x=1−15).

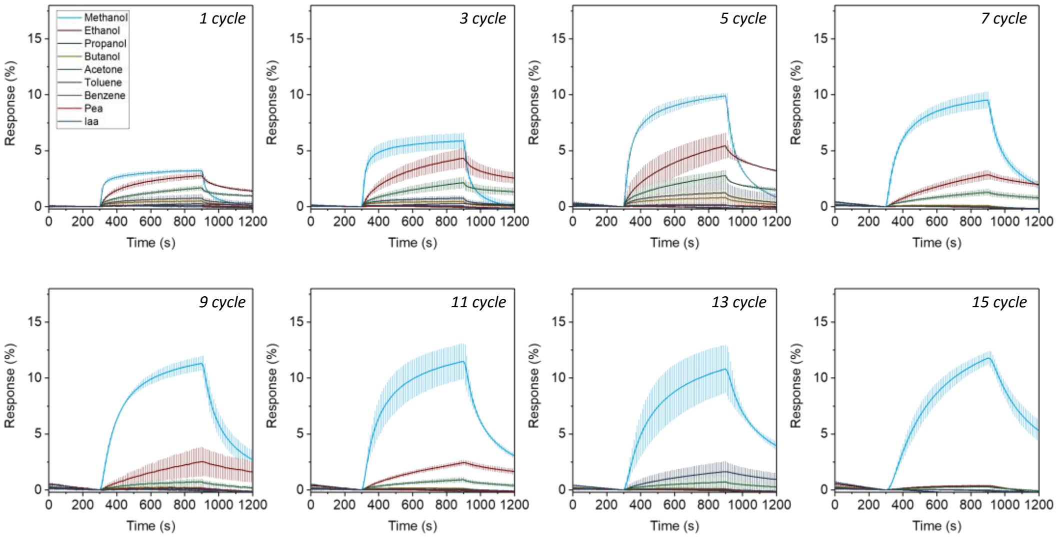

Figure S15. Gas sensing transients of CNT@Mg-HHTT-*x*C (*x*=1, 3, 5, 7, 9, 11, 13, and 15).

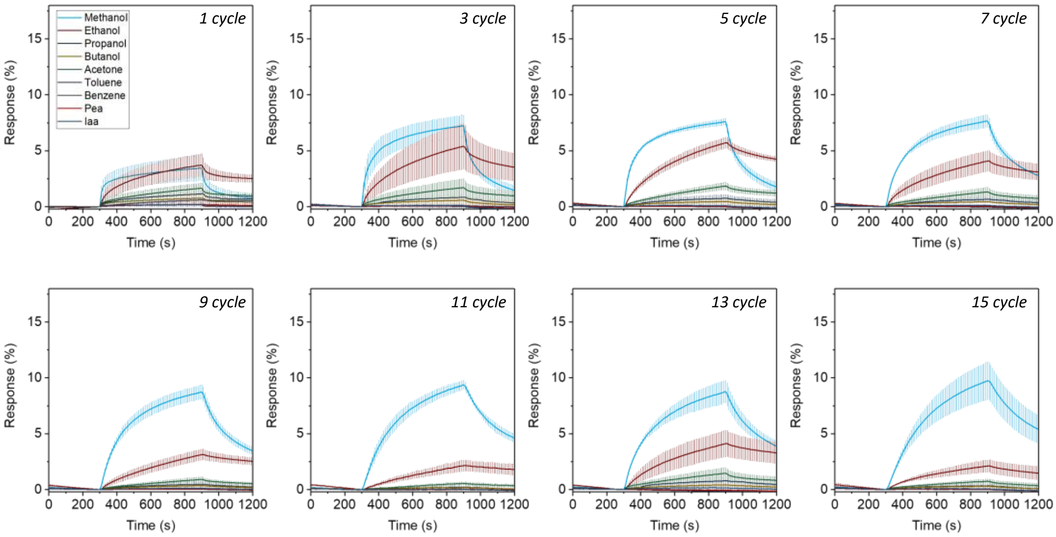

Figure S16. Gas sensing transients of CNT@Ni-HHTT-xC (x=1, 3, 5, 7, 9, 11, 13, and 15).

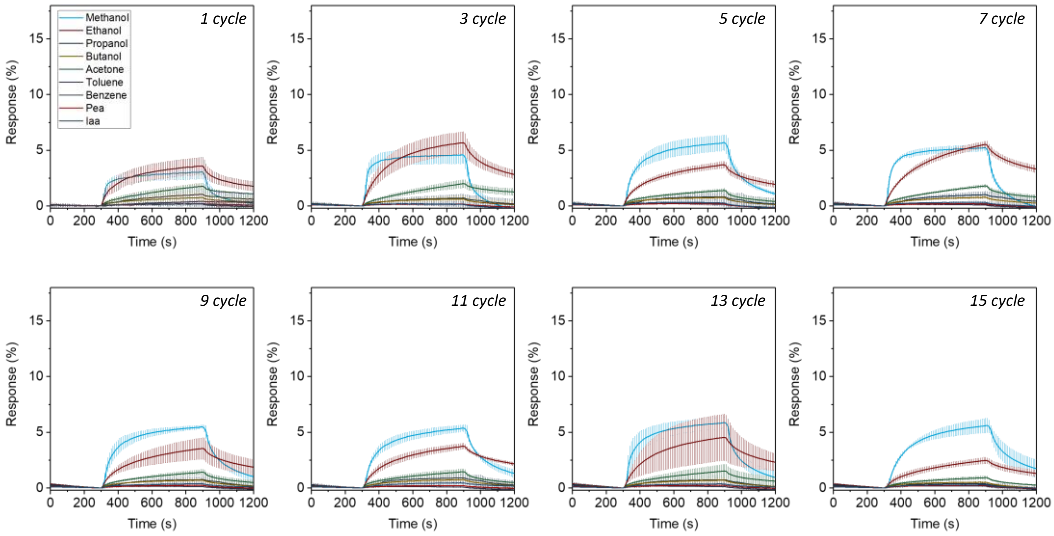

Figure S17. Gas sensing transients of CNT@Cu-HHTT-*x*C (*x*=1, 3, 5, 7, 9, 11, 13, and 15).

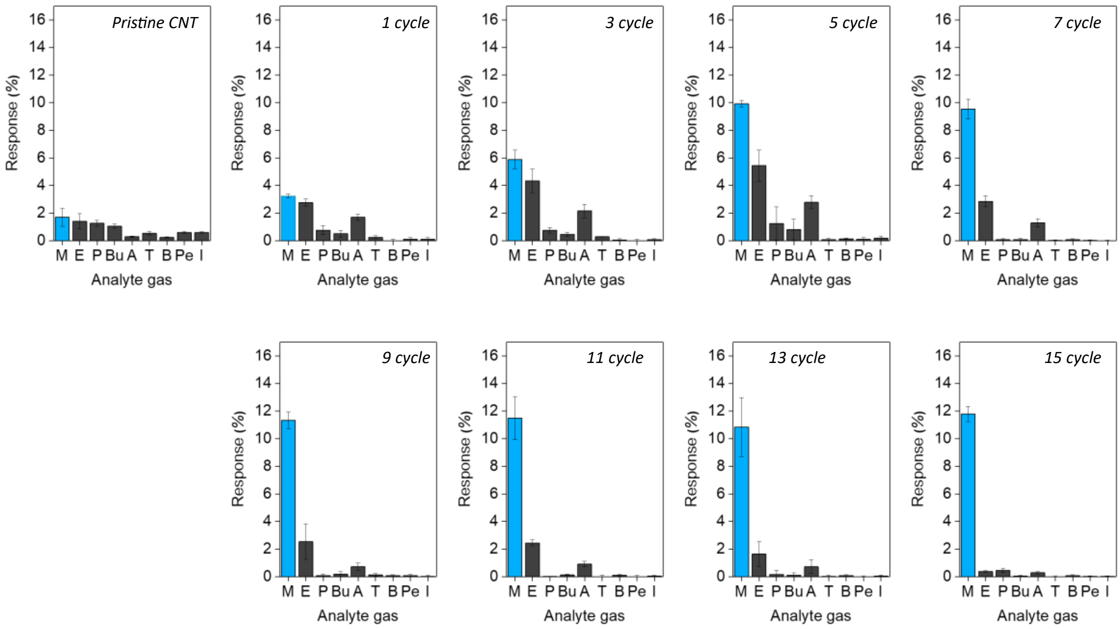

Figure S18. Gas response of CNT@Mg-HHTT-*x*C (*x*=1, 3, 5, 7, 9, 11, 13, and 15).

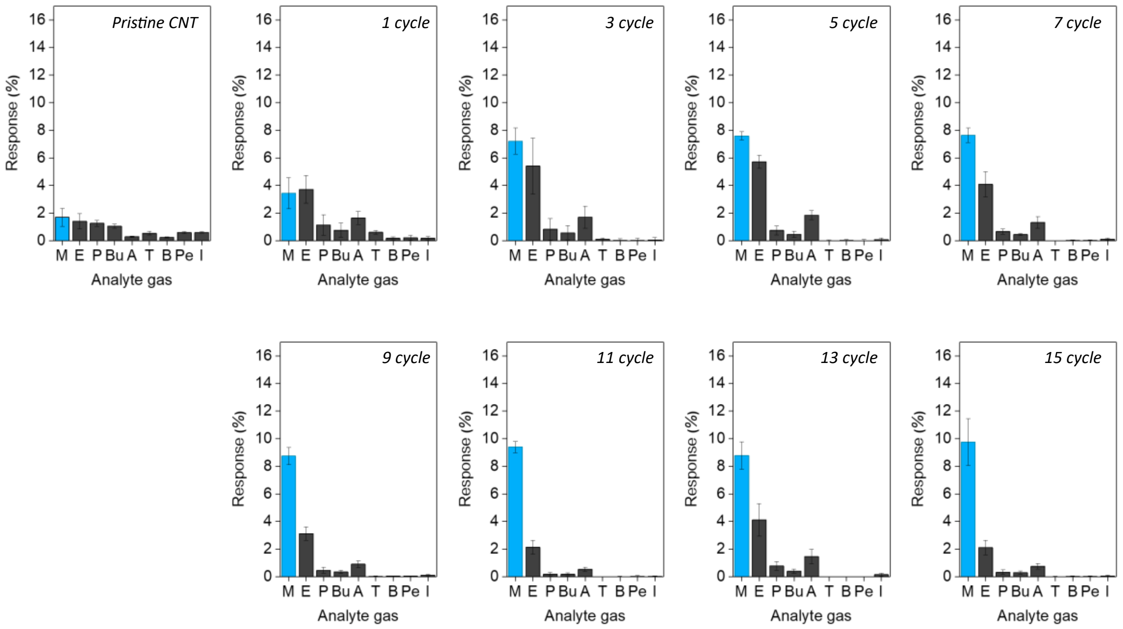

Figure S19. Gas response of CNT@Ni-HHTT-xC (x=1, 3, 5, 7, 9, 11, 13, and 15).

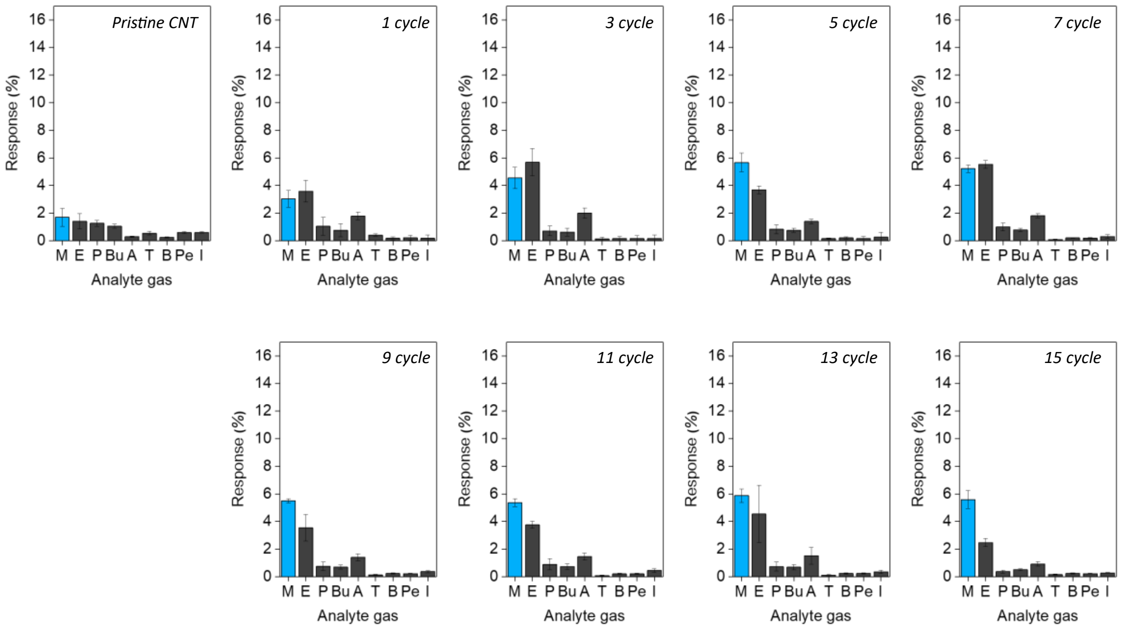

Figure S20. Gas response of CNT@Cu-HHTT-*x*C (*x*=1, 3, 5, 7, 9, 11, 13, and 15).

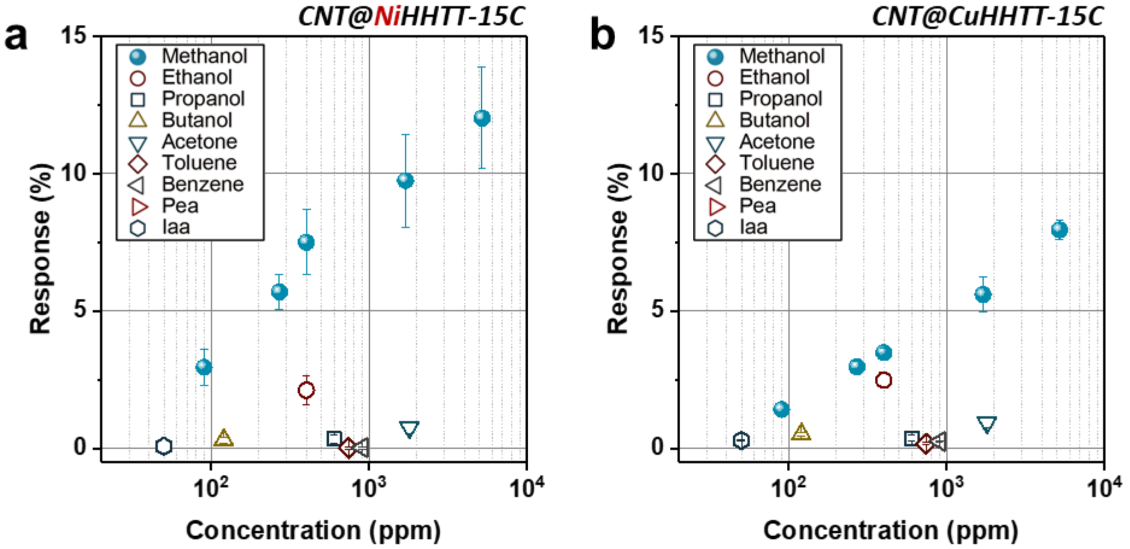

Figure S21. Response of (a) CNT@Ni-HHTT-15C sensors and (b) CNT@Cu-HHTT-15C sensors to 90–5200 ppm of methanol, and to other interference gases at room temperature.

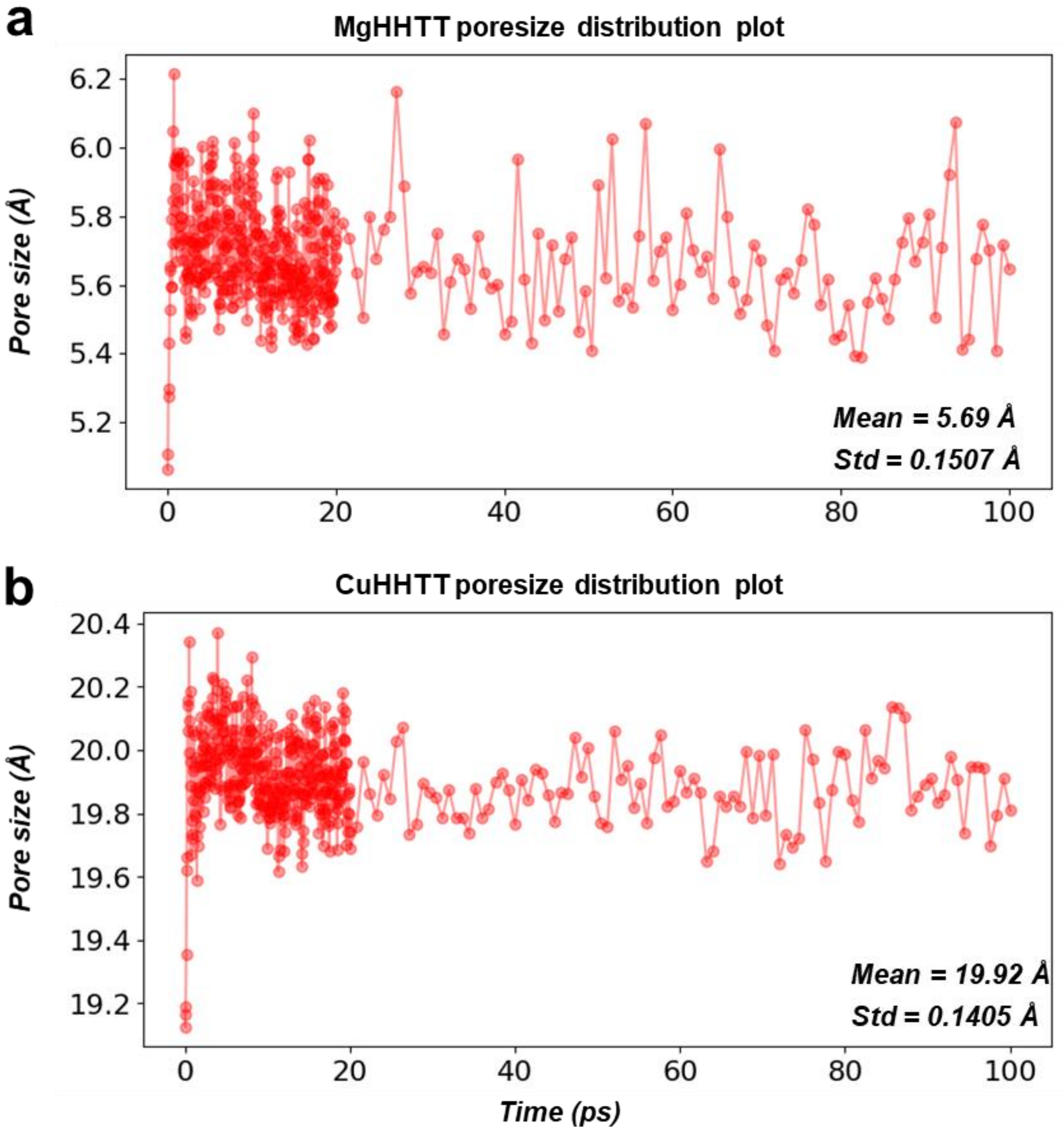

Figure S22. Pore size distribution calculation during 100 ps of simulation time in (a) Mg-HHTT and (b) Cu-HHTT.

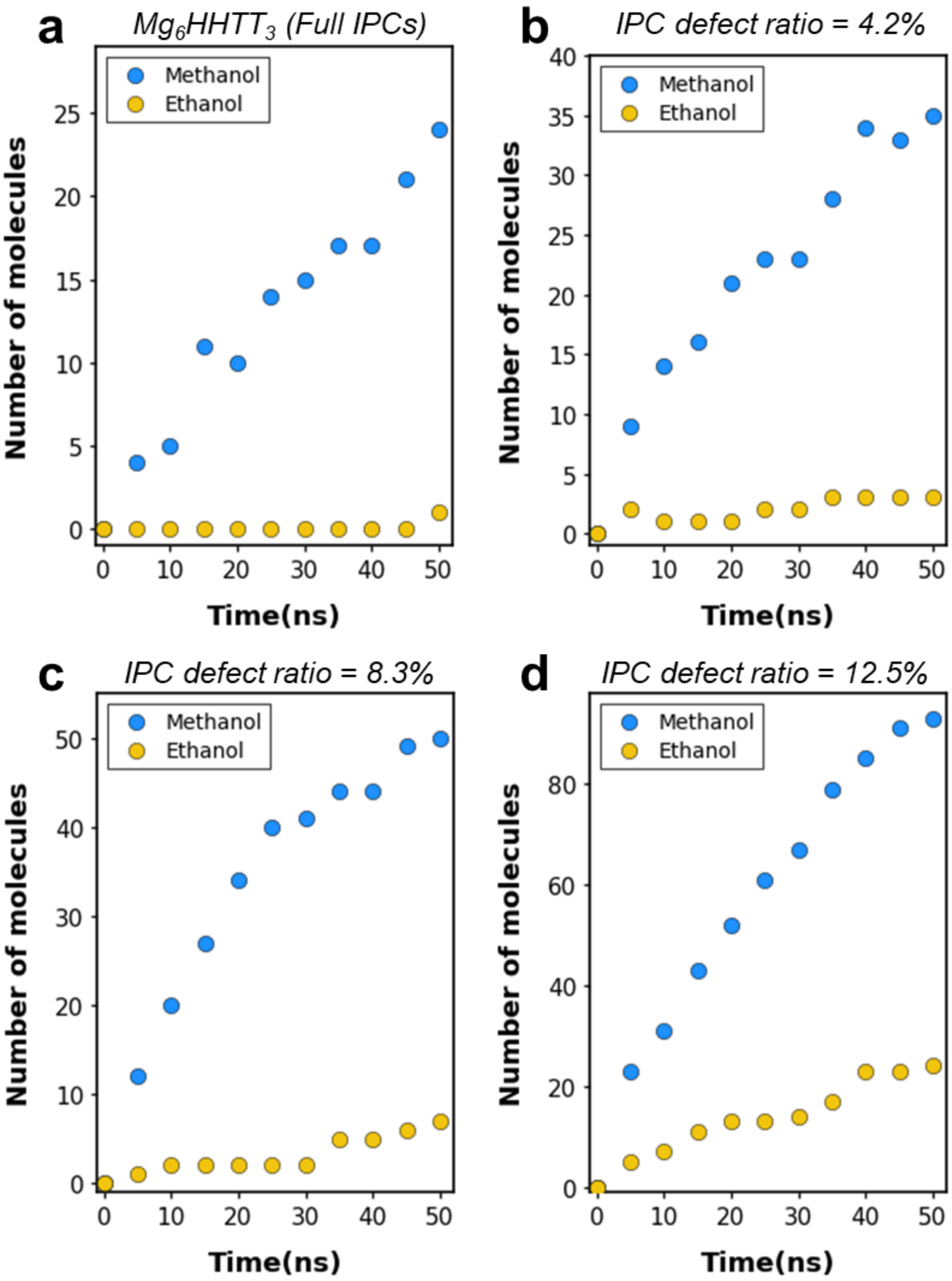

Figure S23. Time-dependent counts of permeating molecules for Mg-HHTT as a function of IPC defect ratio. (a) Reference structure used in the main text. (b) Structure with two IPCs removed (defect ratio = 4.2 %). (c) Structure with four IPCs removed (defect ratio = 8.3 %). (d) Structure with six IPCs removed (defect ratio = 12.5 %).

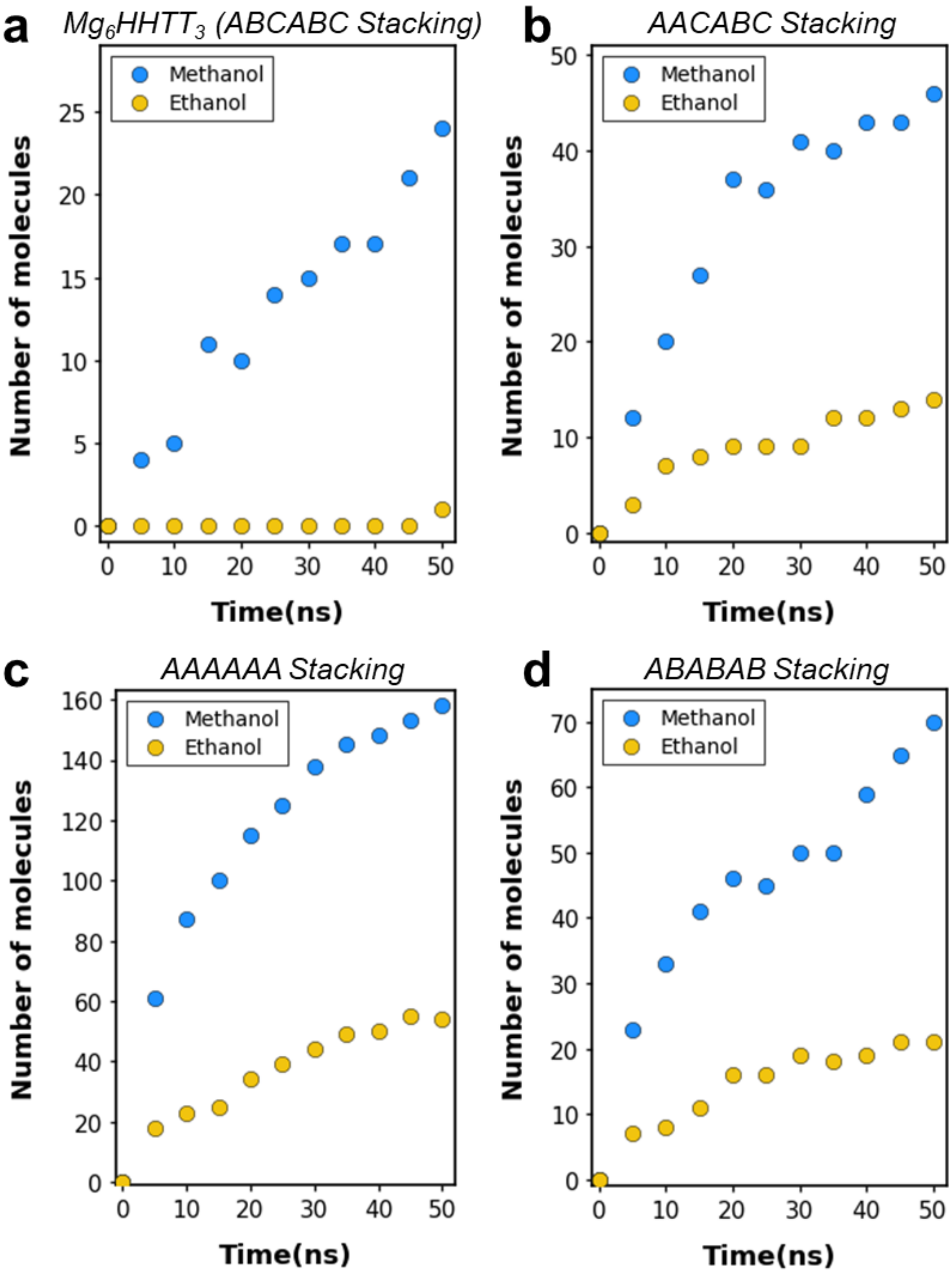

Figure S24. Time-dependent counts of permeating molecules for Mg-HHTT with different stacking order. (a) Reference structure used in the main text. (b) Partially eclipsed AACABC stacking. (c) Fully eclipsed AAAAAA stacking. (d) Slipped-parallel ABABAB stacking.

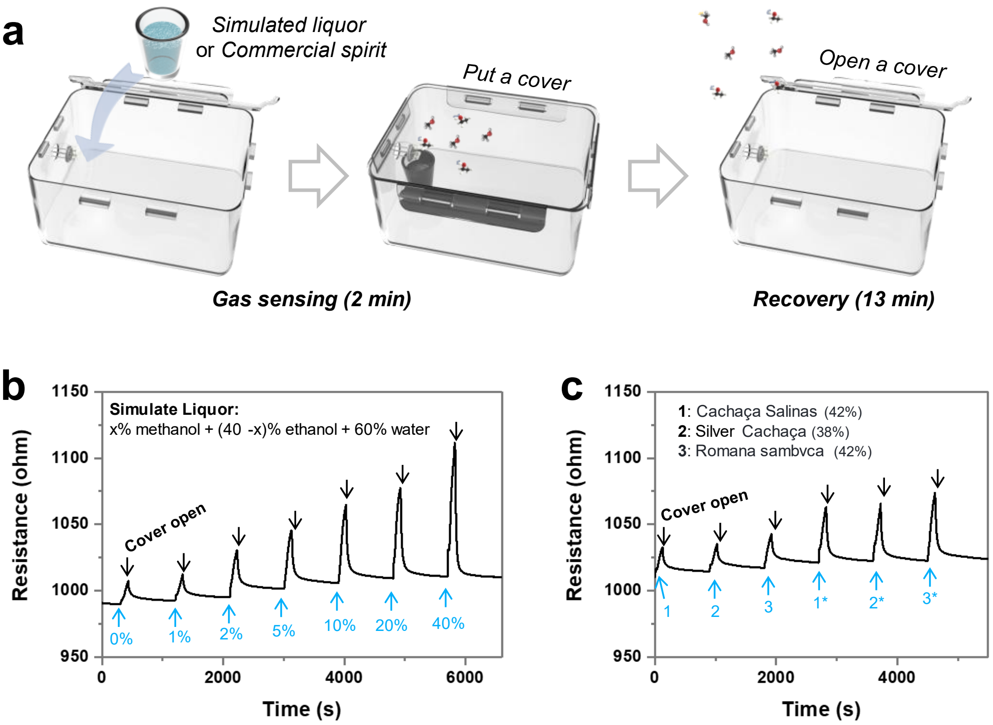

Figure S25. (a) Illustration of the procedure for liquor sensing tests. Gas sensing transients of CNT@Mg-HHTT-15 sensors to (b) methanol-contaminated liquors with methanol concentrations ranging from 0 to 40 vol% at a fixed total alcohol content of 40%, and (c) three commercial spirits—Cachaça Salinas (42%), Silver Cachaça (38%), and Romana Sambuca (42%) (asterisk: 8 vol% methanol added). During the measurements, the sample liquor was placed in a static chamber for 3 min while monitoring the resistance change, followed by removal of the sample and recovery in ambient air. All experiments were conducted under ambient humidity conditions.

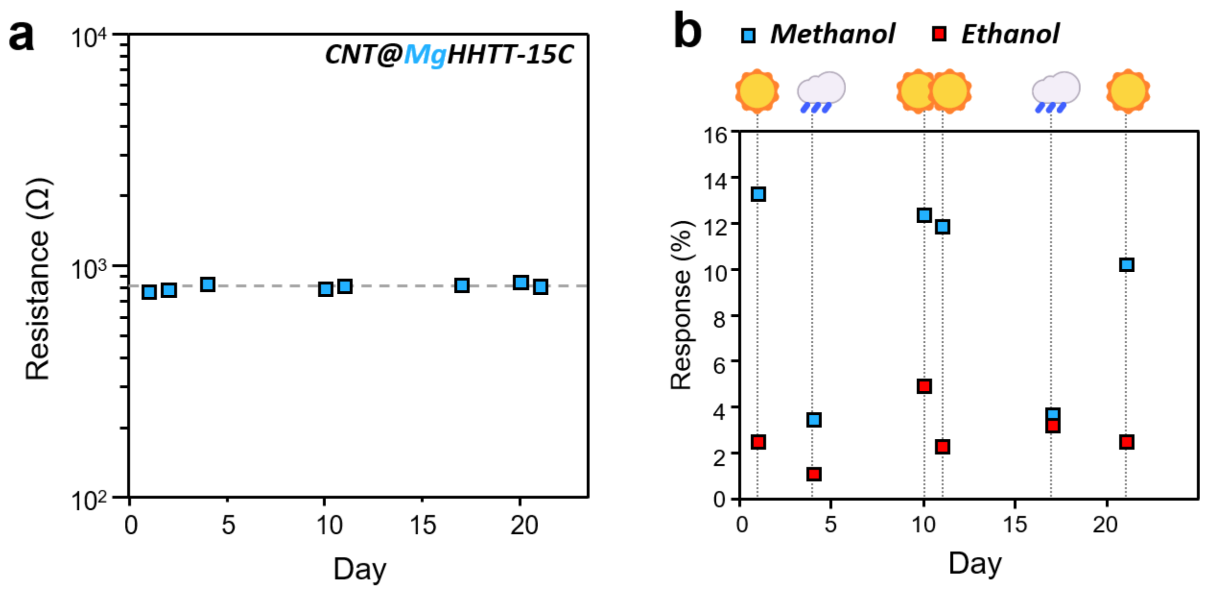

Figure S26. Long-term stability of CNT@Mg-HHTT-15C over 3 weeks under ambient humidity conditions. (a) Resistance, and (b) methanol and ethanol response. The indoor humidity was monitored during the measurements, and prior to each measurement, the sensor was mildly heated at 60 °C for 5 min. Notably, the methanol response decreases sharply under high indoor humidity (rainy conditions; over 50% RH). However, under typical ambient conditions (30–50% RH range), the sensor retains its selectivity toward methanol.

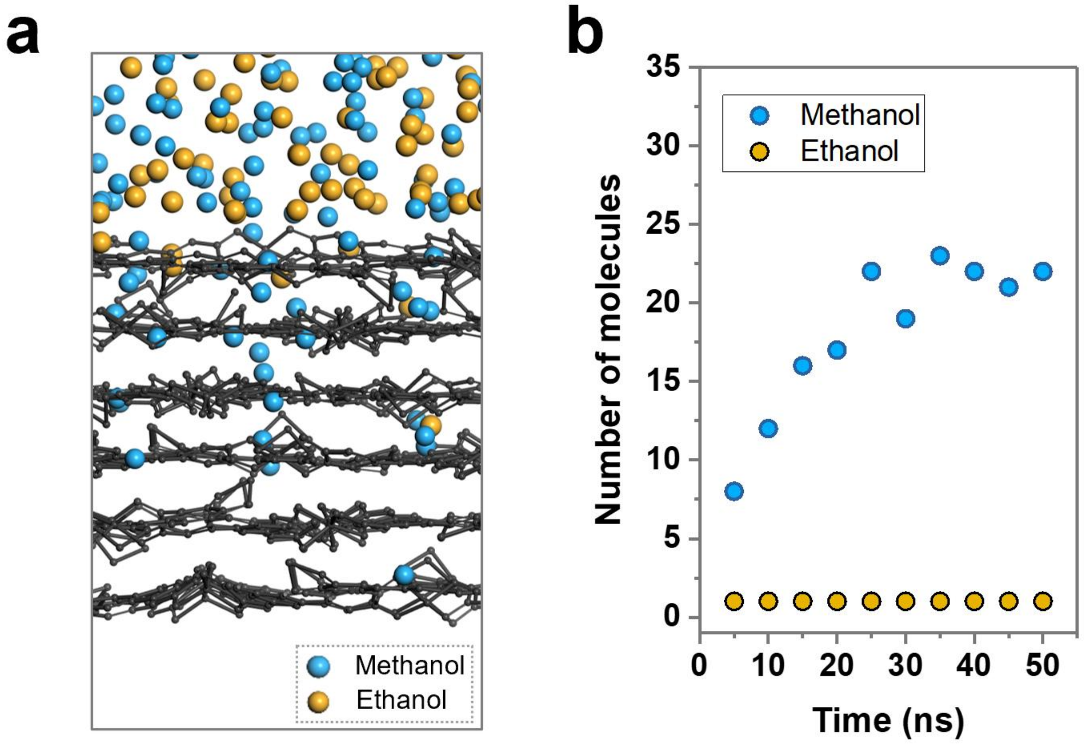

Figure S27. (a) MD simulation image of mixed gases penetration to Mg-HHTT layers. (b) Number of penetrating gases as time varies in Mg-HHTT.

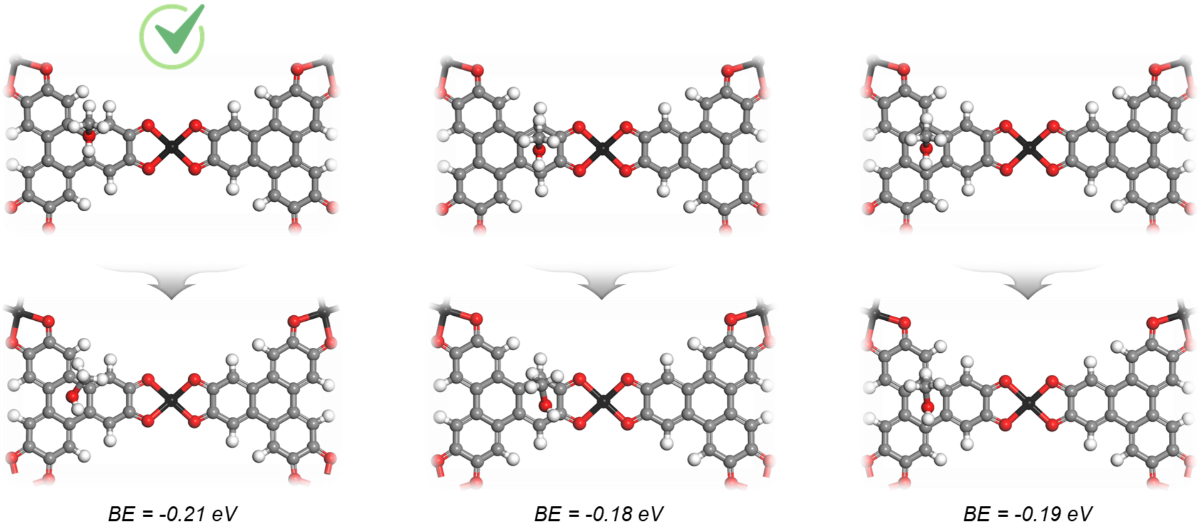

Figure S28. Binding configurations used to determine the adsorption sites between methanol and HHTP linker. The green check mark indicates the most stable structure used in the main text.

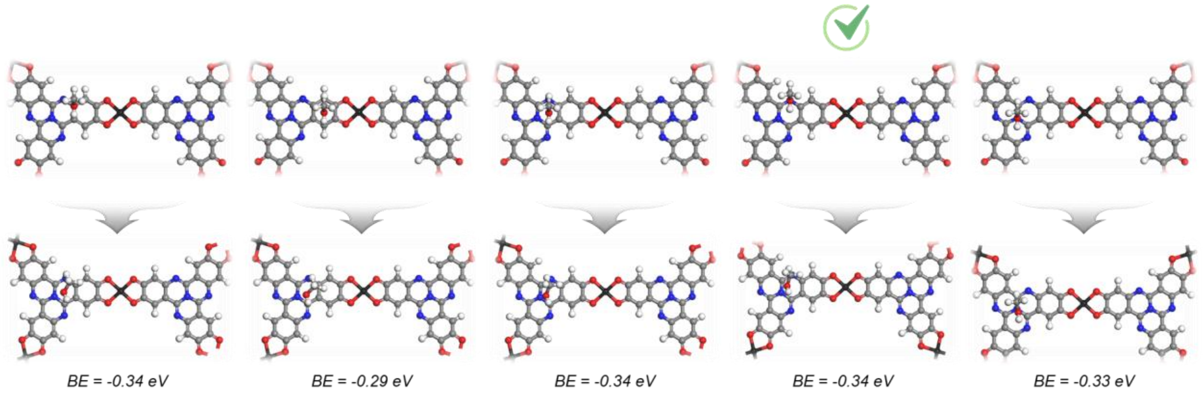

Figure S29. Binding configurations used to determine the adsorption sites between methanol and HHTT linker. The green check mark indicates the most stable structure used in the main text.

[S1] J.-H. Dou, M. Q. Arguilla, Y. Luo, J. Li, W. Zhang, L. Sun, J. L. Mancuso, L. Yang, T. Chen, L. R. Parent, G. Skorupskii, N. J. Libretto, C. Sun, M. C. Yang, P. V. Dip, E. J. Brignole, J. T. Miller, J. Kong, C. H. Hendon, J. Sun, M. Dincă, *Nat. Mater*. **2021**, 20, 222-228.
